# Unique identifiers for small molecules enable rigorous labeling of their atoms

**DOI:** 10.1038/sdata.2017.73

**Published:** 2017-05-23

**Authors:** Hesam Dashti, William M. Westler, John L. Markley, Hamid R. Eghbalnia

**Affiliations:** 1 National Magnetic Resonance Facility at Madison, Department of Biochemistry, University of Wisconsin-Madison, Madison, Wisconsin 53706, USA

**Keywords:** Data integration, Cheminformatics

## Abstract

Rigorous characterization of small organic molecules in terms of their structural and biological properties is vital to biomedical research. The three-dimensional structure of a molecule, its ‘photo ID’, is inefficient for searching and matching tasks. Instead, identifiers play a key role in accessing compound data. Unique and reproducible molecule and atom identifiers are required to ensure the correct cross-referencing of properties associated with compounds archived in databases. The best approach to this requirement is the International Chemical Identifier (InChI). However, the current implementation of InChI fails to provide a complete standard for atom nomenclature, and incorrect use of the InChI standard has resulted in the proliferation of non-unique identifiers. We propose a methodology and associated software tools, named ALATIS, that overcomes these shortcomings. ALATIS is an adaptation of InChI, which operates fully within the InChI convention to provide unique and reproducible molecule and all atom identifiers. ALATIS includes an InChI extension for unique atom labeling of symmetric molecules. ALATIS forms the basis for improving reproducibility and unifying cross-referencing across databases.

## Introduction

Small organic compounds, some classified as ligands^1,2,3,4, 5^, fragments^6,7,8,9, 10^, or metabolites^11,12, 13,14, 15,16, 17^, play prominent roles in biomedical research, for example, in biomarker discovery, screening, and drug discovery^18,19,20^. Numerous databases document the diverse array of structural and functional properties relevant to these compounds^21,22, 23,24, 25,26, 27^, including their chemical properties^28,29^, biological functions, and roles in pathways interaction networks^30,31,32^. These databases provide a substantial volume of unique and as well as overlapping types of content (an up-to-date list of databases related to metabolomics is provided by the Metabolomics Society http://metabolomicssociety.org). Multiple types of experimental data on individual compounds reside in different databases: X-ray structures, nuclear magnetic resonance (NMR) data, mass spectroscopic (MS) data, p*K*_a_ values, melting points, etc. Applications ranging from studies of natural products, to metabolomics, and synthetic chemistry use these heterogeneous data entries from different databases in order to access relevant chemical and physical properties of target molecules and their constituent atoms. It has been proposed that publications link chemical information to persistent open-access databases^33^. However, the validity of the information returned depends on the correct identification of the compound of interest in the publication and in each database, and this requires atomic-scale comparisons. The three-dimensional (3D) specification of the molecule is its photo ID, and it can, in principle, be used as an identifier. However, searches on the basis of 3D structure are unwieldly, and the approach has been to develop unique compound identifiers on the basis of 3D structures. Reproducible unique compound identifiers take into account covalent molecular structure, chirality, and rigorous and complete atom naming. Without the implementation of a unique and reproducible naming and labeling method for relevant compounds, the outcomes of scientific studies can become ill defined. In addition, the retrieval of reliable information relevant to molecules from different databases is dependent on their use of standard unique molecule and atom identifiers. Our investigation has revealed that these requirements for standardized unique molecule- and atom-level identifiers are not fully met in a variety of databases that contain information on organic molecules. These finding have prompted us to investigate approaches to such nomenclature and to propose a solution to their current deficiencies.

Several approaches for creating a reproducible system of assigning unique identifiers to chemical compounds have been proposed. These approaches include the systematic naming method supported by the International Union of Pure and Applied Chemistry (IUPAC https://iupac.org/), the Simplified Molecular-Input Line-Entry System (SMILES)^34,35^, and the International Chemical Identifier (InChI)^36^ developed under the auspices of IUPAC with principal contributions from the U.S. National Institute of Standards and Technology (NIST http://www.nist.gov/) and the InChI Trust (http://www.inchi-trust.org/). One or more instances of these identifiers are currently in use in multiple databases. However, the process of producing unique identifiers, for the purpose of validation, and for creation of cross references between databases, has met with only partial success. The challenge persists of maintaining the consistency and correctness of the identifier-structure-metadata relationship^37^. As a consequence, the aggregation of current knowledge about a molecule remains procedurally complex.

In addition to a unique naming system for chemical compounds, a unique labeling system for the constituent atoms is frequently required. For example, enzymatic reactions are atom specific, and their characterization requires atom-level nomenclature. Atom-specific information generated by quantum mechanical calculations or by experimental data from NMR spectroscopy or X-ray diffraction needs to be associated with standard atom nomenclature. One well-known application of such atom specific data is in NMR metabolic profiling, a method widely used for enhancing our understanding of cellular mechanisms and for identifying metabolic biomarkers^38,39, 40,41, 42,43, 44^. The process of quantifying a metabolite from an 1D-^1^H NMR spectrum requires that NMR peaks from the experiment be matched against those of a reference spectrum, for example from the Biological Magnetic Resonance Data Bank (BMRB^21^) or the Human Metabolome Data Bank (HMDB^22,23,24^). The peaks arising from chemical shifts and spin-spin couplings rely strongly on the structure of the molecule and the sample condition in which the reference NMR spectra of the molecule was collected^45,46^. Full utilization of the reference NMR spectra in metabolic profiling requires the assignment of spectral transitions to the atoms of the molecule.

Our system for unique molecular and atom labeling builds upon the capability of the InChI-1 program, and enhances it further to enable the assignment of unique atom identifiers. We have chosen to use the standard InChI string as the starting point, because (a) the InChI representation is capable of assigning unique identifiers to molecules, (b) this standard is supported by IUPAC and NIST, (c) databases have the corresponding tags for storing this standard, and (d) in contrast to the IUPAC naming system, InChI strings are machine-readable and support mapping between the covalent structure of a molecule and its unique identifier. The enabling feature that allows computer programs to map identifiers to 3D structures is of practical importance for high-throughput processing of information from databases. Our approach, which is named ALATIS (for Atom Label Assignment Tool using InChI String), is embodied in a publicly-available webserver located at (http://alatis.nmrfam.wisc.edu/).

## Results

Application of the ALATIS algorithm to several data sources (see ‘Data sources’ in the Methods section) demonstrates that ALATIS generates unique molecule and atom identifiers in a robust and reproducible format. The complete content of BMRB^21^ and HMDB^22,23, 24^ relevant to metabolite entries provides a full demonstration of ALATIS. The entries in BMRB and HMDB cross reference entries from the PubChem^28^ database; therefore, ALATIS was also used to analyze the PubChem entries to illustrate its ability to validate cross referencing, and to uncover errors. Furthermore, we have used the RCSB PDB Ligand Expo database (http://ligand-expo.rcsb.org/) to demonstrate the versatility of ALATIS. BMRB and RCSB PDB are branches of the Worldwide Protein Data Bank (wwPDB)^47^. We summarize the five key capabilities of ALATIS below; Supplementary Information 1 contains additional details.

### Detection and correction of errors in InChI strings

Of the approximately 60,000 data entries from the target databases, the output of ALATIS identified more than 11,000 entries that contained inaccurate InChI strings. The majority of these inaccurate InChI strings were the result of missing stereochemical information (other factors are described in Supplementary Information 1). ALATIS provided correct and unique InChI strings for all entries that contained 3D structure files.

### Detection of incompatible atom identifiers and generation of unique identifiers

We examined the atom-specific mapping of ^1^H NMR chemical shifts from 701 small molecules present in both BMRB and HMDB. Of the 701 molecules, 552 had incompatible atom labels, which made the comparison of atom-specific resonance data impractical. ALATIS provided unique atom labeling for all entries from both databases as needed to enable accurate assimilations and comparisons across the databases.

### Detection and remediation of incorrect cross references

ALATIS analyzed the currently provided cross references from BMRB and HMDB entries to PubChem entries. By comparing the unique molecule and atom identifiers of the entries corresponding to every cross reference, ALATIS succeeded in identifying and flagging inaccurate cross references. More than 21% of the current cross references were found to be inaccurate. As described in Supplementary Information 1, the inconsistency of information deposited in databases is the primary reason for the high percentage of inaccurate cross references. We have divided the inaccurate cross references into five categories as detailed in Supplementary Information 1. These categories, and the accompanying examples, highlight the central role of unique identifiers, and further emphasize the critical importance of validating cross references between databases in order to mitigate the use of potentially misleading information. The provision of validated unique identifiers will be a critical enabling factor in the coming era of federated databases. We have demonstrated here that our method based on the InChI string and ALATIS software can serve this purpose.

### Creation of new and unique cross references between entries of different databases

The utility of ALATIS in producing new cross references is not limited to metabolites. Analysis by ALATIS of the RCSB PDB Ligand Expo revealed numerous inaccuracies (outlined in Supplementary Information 1). In addition, from the 22,758 entries in the Ligand Expo, ALATIS created 863 new cross-references to entries in BMRB, 1,693 new cross references to entries in HMDB, and 1985 new cross references to entries in PubChem. The procedures used for generating these cross-references and the outcomes are explained in Supplementary Information 1.

### Creation of unique identifiers for mixtures

ALATIS uses the information layers in the standard InChI string to delaminate and identify the individual molecules in a mixture. Every molecule in the mixture is associated with a block of indices that together generate a unique identifier that specifies the appearance and arrangement of the individual constituents. The details of this procedure are provided in Supplementary Information 2.

## Discussion

The number of chemical and biomedical investigations involving organic molecules has greatly increased in recent years. As a result, the demand for seamless access to relevant information from databases has increased correspondingly. The federation of databases offers an approach to making such information more readily available. However, as we have shown, this demands that unique and reproducible identifiers be used for molecules and their constituent atoms. Our emphasis on the additional requirement for atomic-specific labels is inspired by the growing number of applications that rely on these. We have demonstrated that the standard International Chemical Identifier (InChI) string as further elaborated under current rules has the richness needed for this task. We have shown that by utilizing the standard identifier mechanism, we are able to produce a unique atom-labeling system that can be used for creating and validating cross references between databases.

We have developed a software package that implements this methodology by taking advantage of the InChI-1 software program for the generation of standard InChI strings. We have demonstrated applications of ALATIS for the analysis of four major small-molecule databases: BMRB, HMDB, RCSB PDB Ligand Expo, and PubChem. The outcomes of these analyses are available on our webserver. We have shown that ALATIS can be utilized for maintaining uniqueness across a database, and therefore can be used for validation and creation of cross references between databases. In addition, we have demonstrated that ALATIS can be used to produce unique and reproducible atom identifiers. The ALATIS software package has been incorporated recently into the BMRB pipeline for its new small molecule entries, and it is planned to use this approach to remediate the entire small molecule database at BMRB. In addition, we have made ALATIS available for remediation of the RCSB PDB Ligand Expo. ALATIS is available for public use on a webserver at NMRFAM (http://alatis.nmrfam.wisc.edu/) and is embedded into the NMRbox^48^ (https://nmrbox.org/).

## Methods

### Requirements for the construction of Molecular identifiers

The preferred IUPAC name (PIN) has been introduced as a systematic naming of chemical compounds to provide a unique and human readable naming system^49^. However, this naming system is proprietary and rarely used in public databases. Other IUPAC names (e.g., IUPAC traditional, IUPAC CAS, IUPAC OpenEye, IUPAC systematic) are favored in practice. These dominantly used IUPAC identifiers allow for the assignment of multiple correct names to the same compound. For example, the compound toluene has synonyms phenylmethane and methylbenzene. Further complicating uniqueness is the problem that, by the use of IUPAC names, it is possible to assign one name to multiple compounds: for example, both scyllo-inositol and myo-inositol can be called cyclohexane-1,2,3,4,5,6-hexol. More generally, families of symmetric compounds that differ in their stereochemistry can suffer this non-uniqueness problem. Moreover, the IUPAC naming system lacks machine readability: a computer program (without having a complete dictionary of IUPAC names and three-dimensional structures) cannot identify the atoms and their corresponding bonds for a molecule of interest from its IUPAC name. In addition to the deficiencies of this procedure, databases often fail to use the correct IUPAC naming system. An example of improper use of these systematic names can be seen in the PubChem entries for beta-Ala-3-methyl-His (Data Citation 1) and anserine (Data Citation 2), which describe the same molecule. The IUPAC name for the (Data Citation 1) ‘2-(3-aminopropanoylamino)-3-(3-methylimidazol-4-yl)propanoic acid’ carries no information about the stereochemistry of the molecule, whereas the entry for anserine contains the correct and complete IUPAC name ‘(2S)-2-(3-aminopropanoylamino)-3-(3-methylimidazol-4-yl)propanoic acid’. This compound exists in both R and S isomers, and disregard of the stereochemistry results in the incorrect representation of the molecule. Another example of the improper use of the IUPAC names as synonyms can be seen in the HMDB^22,23,24^ entry for furan (Data Citation 3), which reports ‘1-alpha-D-glucopyranosyl-2-beta-D-fructofuranoside’ (data extracted from HMDB on August 18, 2016) as a synonym, although it actually is a completely different molecule (sucrose). These examples identify methodological problems that stem from the use of conventional IUPAC naming systems.

Alternatively, the SMILES naming system is widely accepted, partly owing to the human- and machine-readability of SMILES strings^50,51, 52^. However, it is well known that non-canonical SMILES strings fail to provide unique one-to-one compound identifiers. The commonly cited example is ethanol, which can be represented by SMILES strings ‘CCO’ and ‘C(C)O’. As a result, a number of different algorithms for canonicalization of SMILES strings have been developed (for comprehensive discussions see refs 53,54). These algorithms produce a unique SMILES string for a molecule, but different canonicalization algorithms are likely to produce different strings. The community-organized OpenSMILES website (http://opensmiles.org/opensmiles.html#canonicalization) notes that the canonicalization of SMILES strings can be helpful for searching within a database but not between databases that may utilize different canonicalization algorithms. For example, phosphonoacetic acid (C2H5O5P) (Data Citation 4, BMRB^21^ SMILES: ‘C(C(=O)O)P(=O)(O)O’) can be represented by at least two different canonical SMILES strings (‘O=C(O)CP(=O)(O)O’ and ‘OC(=O)CP(O)(O)=O’); one generated by MolConvert (from ChemAxon; see acknowledgment for citation), and another by RDKit (open-source cheminformatics software http://www.rdkit.org/) software package. Multiple canonical identifiers present a challenging non-uniqueness problem, which hinders the creation of valid cross references between databases.

Another convention is the InChI naming system, which provides a unique representation for chemical compounds^36^. The InChI-1 software program (http://www.inchi-trust.org/download/104/InChI_TechMan.pdf) from the InChI Trust generates a single standard string for a compound, and every standard InChI string corresponds to only one compound^36^. This unique one-to-one mapping of InChI between molecules and identifiers has inspired another canonicalization of the SMILES representation based on the corresponding InChI string of the molecule^54^. However, this new ‘InChIfied’-SMILES identifier is not widely used, and the software package encoding this method (Open Babel^55^) is incapable of producing complete SMILES strings (to our knowledge, hydrogen atoms are not considered in the latest version 2.3.2 of the software package). Although InChI can be unique, in principle, we show that, in practice, variant uses of this naming system are found in different databases. As a result, uniqueness is compromised (as noted in the Results Section), and hence the ability to identify and access all relevant data may be lost. However, the adaptation of InChI, as implemented by ALATIS, is capable of assigning unique molecule and atom identifiers across databases.

### Requirements for unique labeling of atoms

**Figure 1:**
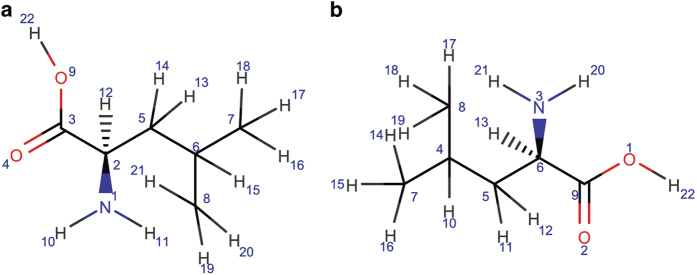
Non-unique labeling of atoms in databases. (**a**) 2D representation of the structure file of L-leucine (Data Citation 5) downloaded from BMRB^21^. (**b**) 2D representation of the structure file of L-leucine (Data Citation 6) downloaded from HMDB^22,23,24^. As shown in the diagram the two structural representations utilize different atom numberings. As a result, the relationship between atoms and their labels (numbers) is database-specific.

The following example illustrates the importance of unique atom labeling. In this example the database entries corresponding to L-leucine from BMRB (Data Citation 5) and HMDB (Data Citation 6) are used. Both entries report 1D ^1^H NMR chemical shifts for the compound. The atom labeling schemes used by two databases are entirely different (Fig. 1). As a result, the chemical shift assignments to particular atoms is database-dependent. Database-dependent atom naming schemes serve as a roadblock to database federation. Therefore, in addition to a unique identifier for each molecule, it is important to be able to uniquely and reproducibly label all of its constituent atoms across databases.

### Overview of the process used to create unique and reproducible identifiers

**Figure 2:**
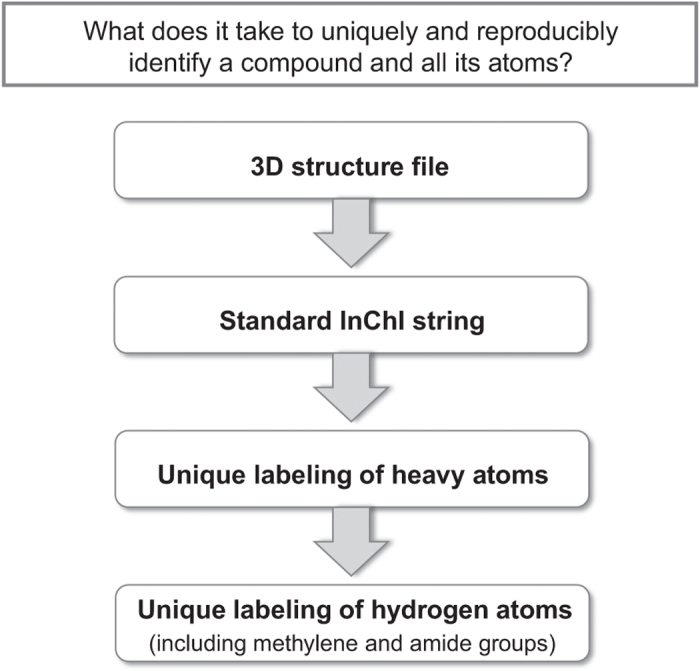
Process for creating a unique and reproducible molecular identifier and complete atom labels. Overview of the steps considered in ALATIS.

The multiplicity of names and synonyms, and the task of verifying the validity of their assignments to molecules in different databases, as well as the lack of unique molecule and atom identifiers, presents a fundamental challenge for data management of chemical compounds in databases. These challenges, explained above in the Results Section, can be addressed by a system that creates an identifier that is unique for the molecule along with unique and reproducible labeling of all atoms within the molecule. Figure 2 shows the steps taken in our methodology for creating such identifiers.

**Figure 3:**
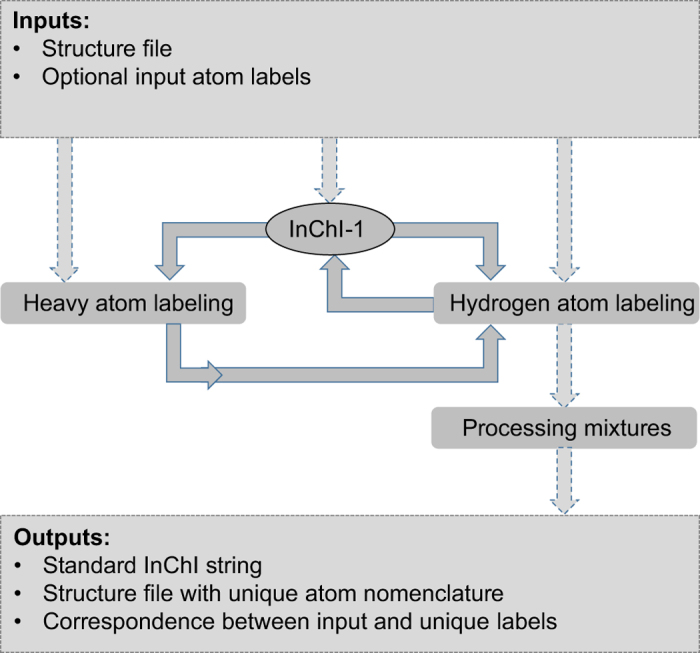
Flowchart for the software package ALATIS. The webserver for ALATIS accepts a structure file for the compound as input (SDF or MDL Mol-V2000 file) along with optional atom labels. Three modules receive the data (dashed arrows). The InChI-1 program executes in the background to generate the standard InChI string for the input. The modules work in concert in order to assign unique labels to heavy atoms as well as to hydrogen atoms of the molecule (solid arrows). To label heavy atoms, two sub-modules are used to construct two graph representations for the molecule (using the input structure file and the generated standard InChI strings; see Supplementary Information 2 for the details of the graph representation). Another sub-module maps the graphs to a representation suitable for assigning unique labels to the heavy atoms. The module responsible for assigning unique labels to the hydrogen atoms imposes temporary chiral centers on the heavy atoms in order to distinguish between the hydrogens attached to each heavy atom. The idea of introducing temporary chiral centers is elaborated further to accommodate atom labeling of symmetric molecules. During this process the InChI-1 program is executed repeatedly and iteratively (solid arrows). In the cases where the input structure file contains multiple molecular structures (for example representing different tautomeric states), a separate module carries out the processing. ALATIS reports unique labels for molecules in the mixture and their constituent atoms. ALATIS outputs a standard InChI string for the compound, a structure file that contains the unique labels of the atoms, and a map between the atoms labels of the input and the generated unique atom labels.

The key to producing unique and reproducible compound and atom identifiers is a 3D structure file, because it offers a complete representation of the molecule. Given the 3D structure file, ALATIS follows a strict protocol to derive the unique identifiers. As outlined in Fig. 3, this protocol consists of three main modules that (a) label heavy atoms, (b) label hydrogen atoms with special considerations for chiral, prochiral, and primary amide centers of symmetric and asymmetric molecules, and (c) label all atoms in mixtures of molecules. Supplementary Information 2 describes this protocol in detail.

### Code availability

ALATIS is available to the public as a web-service via our web-server (http://alatis.nmrfam.wisc.edu/), and also through the NMRBox virtual machine. The custom source code, developed using MATLAB in the Linux environment (MATLAB 2016a for CentOS 6.5), is available upon request from the corresponding authors. This work is copyrighted under the terms of GPL. The web-service and the source codes are provided on an ‘as is’ basis without warranty of any kind, either expressed or implied. Any usage of the web-server, or modification and application of the source codes are free for academic use when this publication is cited.

### Data sources

This section highlights steps used in downloading and preprocessing of all compounds contained in the four test databases: BMRB, HMDB, PubChem, and RCSB PDB Ligand Expo. All of the description files (NMR-STAR and xml) and structure files (Mol and SDF) were downloaded on August 18, 2016.

#### BMRB:

1. Repository name: Biological Magnetic Resonance Databank^21^
2. Database Identifier: http://www.biosharing.org/biodbcore-000584
3. Publication year: 2008
4. Processing: entire BMRB entries were downloaded in NMR-STAR format from the ftp service of the BMRB website. The NMR-STAR files were processed using the PyNMRSTAR (https://github.com/uwbmrb/PyNMRSTAR/) program to extract the deposited InChI strings for every compound (the tag ‘_Chem_comp.InCHi_code’ in the NMR-STAR files) and also the tag ‘_Chem_comp.Struct_file_name’ was used to construct a hyperlink to the corresponding structure file for every entry. In order to extract the cross references from BMRB entries to PubChem entries, the NMR-STAR tag ‘_Chem_comp_db_link’ that cited the PubChem compound ID was isolated (CID was used exclusively, and PubChem substance IDs were discarded).

#### HMDB:

1. Repository name: The Human Metabolome Database^22–24^
2. Database Identifier: https://biosharing.org/biodbcore-000552
3. Publication year: 2007
4. Processing: entire HMDB data entries and structure files were downloaded from HMDB’s download webpage (http://www.hmdb.ca/downloads); these data were found to differ somewhat from the entries available on the HMDB web pages. Text-processing modules were used to extract the InChI strings and PubChem citation of these compounds by processing the HMDB’s xml files using the tags ‘<inchi>’ and ‘<pubchem_compound_id>’, respectively.

#### PubChem:

1. Repository name: PubChem^28^
2. Database Identifier: https://biosharing.org/biodbcore-000455
3. Publication year: 2004
4. Processing: the PubChem Download Service was used to download PubChem compound IDs, the corresponding PubChem entries (xml formatted), and their structure files. The InChI string for each compound was extracted by parsing the corresponding PubChem xml file using the tag ‘<PC-InfoData_value_sval>’.

#### RCSB PDB Ligand Expo:

1. Repository name: Ligand Expo
2. Database identifier: https://biosharing.org/biodbcore-000510
3. Processing: the ligand Expo database was downloaded from the Download web-page of its web-server (http://ligand-expo.rcsb.org/). Two compact files were downloaded from this page that correspond to the InChI (tab delimited text) and the structure file (SDF/MOL format) of the compounds in the database. In-house text-processing modules were used to process these files and extract the InChI and structure file for every compound.

## Additional Information

**How to cite this article:** Dashti, H. *et al.* Unique identifiers for small molecules enable rigorous labeling of their atoms. *Sci. Data* 4:170073 doi: 10.1038/sdata.2017.73 (2017).

**Publisher’s note:** Springer Nature remains neutral with regard to jurisdictional claims in published maps and institutional affiliations.

## Supplementary Material

Supplementary Information
