# Supplementary material for: Unique identifiers for small molecules enable rigorous labeling of their atoms
Source: Sci Data. 2017 May 23;4:170073. doi: 10.1038/sdata.2017.73 (PMC5441290; doi:10.1038/sdata.2017.73)
Supplement: Supplementary Information [file sdata201773-s1.pdf]

Supporting Information

Unique identifiers for small molecules enable rigorous labeling of their atoms

Hesam Dashti, William M. Westler, John L. Markley, Hamid R. Eghbalnia

National Magnetic Resonance Facility at Madison, Department of Biochemistry, University of Wisconsin-Madison, WI, USA

Table of contents

		Page
Supporting Information 1: Demonstration of applications of ALATIS		2
I.	ALATIS analysis uncovered errors in many deposited InChI strings	2
II.	ALATIS analysis demonstrates the need for standardization of unique compound identifiers and atom labels	6
III.	Use of ALATIS to validate cross references	7
IV.	Use of ALATIS in creating cross references between databases	15
	References	16
Supporting Information 2: Unique label assignment to all atoms		17
I.	Unique labeling of heavy atoms	17
II.	Unique labeling of hydrogen atoms	20
III.	Unique labeling of all atoms in a mixture of compounds	25
	References	27

Supporting Information 1

Results: Demonstration of applications of ALATIS

I. ALATIS analysis uncovered errors in many deposited InChI strings

Standard InChI strings are currently used in databases as unique molecular identifiers. To validate the correctness of these identifiers, we used ALATIS to evaluate the deposited InChI strings against InChI strings derived from their corresponding molecular structure files by the InChI-1 program. All entries in the target databases (see Data sources) were processed by using an automated procedure developed in-house. As indicated in Table 1, the three target databases (BMRB, HMDB, PubChem) were found to contain a large number of incorrect InChI strings. Those entries lacking a structure file could not be validated.

Table 1. Analysis of InChI strings in databases [a]

	BMRB	PubChem	HMDB
Number of entries examined	1456	17207	41497
Number of entries lacking a structure file	4	0	72
Number of entries with incorrect InChI strings [b]	614	3529	7828
Number of entries with InChI strings flagged as non-standard	447	0	7602
Number of entries with InChI strings having an improper stereochemistry layer	594	3529	7818

[a] Carried out on August 18, 2016

[b] As indicated by mismatch between the InChI string in the entry and one derived by the InChI-1 program from the corresponding molecular structure file.

The most common reason for an incorrect InChI string was an improper stereochemistry layer. The InChI stereochemistry layer represents the chirality of the atoms - potentially indicating distinct compounds with different chemical properties. The second most common reason for error was a non-standard InChI string. A third reason for errors was mismatch between the InChI string and its corresponding structure file. For example, the InChI string in BMRB entry bmse000465 corresponds to two tautomeric forms of phthalaldehydic acid, whereas the structure file represents a single tautomer (Figure 1 a,b). In our analysis of HMDB, the majority of errors resulted from the incompleteness of deposited structure files; many of which gave only a 2D representation of the molecule. (We note that many data entries in HMDB have incorporated 3D structures files subsequent to our analysis.) An example of a discrepancy between the deposited structure file and the deposited InChI string resulting from lack of a 3D structure file is shown in Figure 1 c,d; note that the two structures do not match. In our analysis of PubChem, all detected errors were the result of an improper stereochemistry layer. An example is shown in Figure 1 e: the deposited InChI string for 1-ipomeanol in PubChem entry CID 161819 lacked the stereochemistry layer ('/t') present in the InChI string generated from the structure file in the entry by the InChI-1 program.

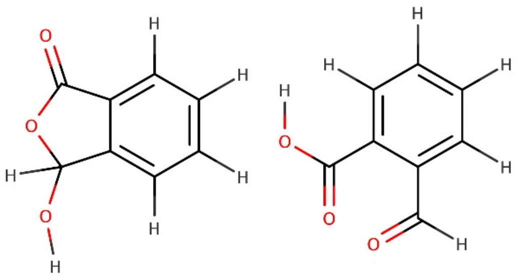

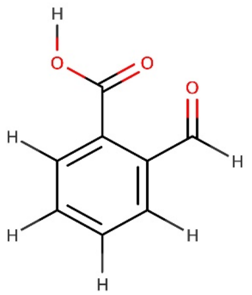

(a) Deposited InChI: InChI=1S/2C8H6O3/c9-7-5-3-1-2-4-6(5)8(10)11-7;9-5-6-3-1-2-4-7(6)8(10)11/h1-4,7,9H;1-5H,(H,10,11)

(b) Standard InChI: InChI=1S/C8H6O3/c9-5-6-3-1-2-4-7(6)8(10)11/h1-5H,(H,10,11)

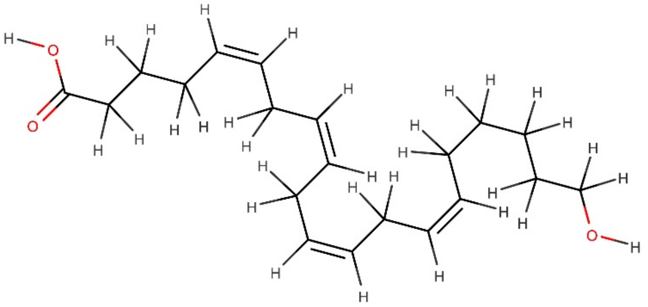

(c) Deposited InChI: InChI=1S/C20H32O3/c21-19-17-15-13-11-9-7-5-3-1-2-4-6-8-10-12-14-16-18-20(22)23/h1,3-4,6-7,9-10,12,21H,2,5,8,11,13-19H2,(H,22,23)/b3-1-,6-4-,9-7-,12-10-

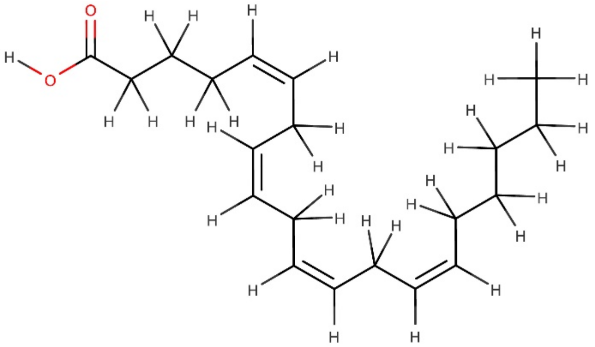

(d) Standard InChI: InChI=1S/C20H32O2/c1-2-3-4-5-6-7-8-9-10-11-12-13-14-15-16-17-18-19-20(21)22/h6-7,9-10,12-13,15-16H,2-5,8,11,14,17-19H2,1H3,(H,21,22)/b7-6-,10-9-,13-12-,16-15-

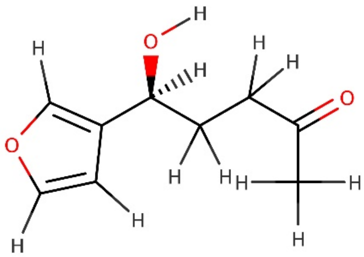

(e) Deposited InChI: InChI=1S/C9H12O3/c1-7(10)2-3-9(11)8-4-5-12-6-8/h4-6,9,11H,2-3H2,1H3
Standard InChI: InChI=1S/C9H12O3/c1-7(10)2-3-9(11)8-4-5-12-6-8/h4-6,9,11H,2-3H2,1H3/t9-/m0/s1

Figure 1. Mismatch between deposited InChI strings and those generated by the InChI-1 program. (a) The InChI string for phthalaldehydic acid from BMRB entry bmse000465 and the 2D structural representation derived from that InChI string (two tautomeric forms of the molecule). (b) The 2D representation of the molecular structure file from BMRB entry bmse000465 and the standard InChI string generated from that structure file by the InChI-1

program. (c) The InChI string for arachidonate from HMDB entry HMDB60102 and the 2D structural representation of this InChI string. (d) The deposited structure file for arachidonate in HMDB entry HMDB60102 and its corresponding standard InChI string (generated from the molecular structure file by the InChI-1 program). Note that the number of atoms in (c) and (d) do not match; (c) has an extra oxygen atom. (e) The deposited InChI string for 1-ipomeanol in PubChem (CID 161819) compared with the standard InChI generated by the InChI-1 program using the structure of the compound in PubChem. Comparison of the InChI strings reveals that the deposited InChI lacked the stereochemistry layer ('/t').

The rigorous definition of stereochemistry is of critical importance. For example, when their stereochemistry is ignored, D-(+)-glucose and D-(+)-mannose have exactly the same InChI string: InChI=1S/C6H12O6/c7-1-2-3(8)4(9)5(10)6(11)12-2/h2-11H,1H2. Another example that illustrates problems caused neglect of stereochemistry is furnished by scyllo-inositol (C6H12O6, BMRB ID: bmse000113), muco-inositol (C6H12O6, BMRB ID: bmse000102), and myo-inositol (C6H12O6, BMRB ID: bmse000103). These three compounds have different physical and chemical properties, yield distinctly different NMR spectra (shown in Figure 2), and have different standard InChI strings. However, the InChI string in the PubChem entry for myo-inositol (CID: 892) lacks the stereochemistry layer: InChI=1S/C6H12O6/c7-1-2(8)4(10)6(12)5(11)3(1)9/h1-12H. As described by Duchem et al. [1], there are 64 members of inositol family that, based on their symmetry, can be reduced and captured by nine distinct structures as shown in Figure 2. These distinct inositol molecules [1-2] have distinctive stereochemistry layers in their corresponding standard InChI strings. The use of standard InChI strings as an identifier is a requirement for the creation and validation of cross references: a non-standard InChI string fails to distinguish the relative or racemic stereochemistry of the molecule, thus compromising the uniqueness of the identifier.

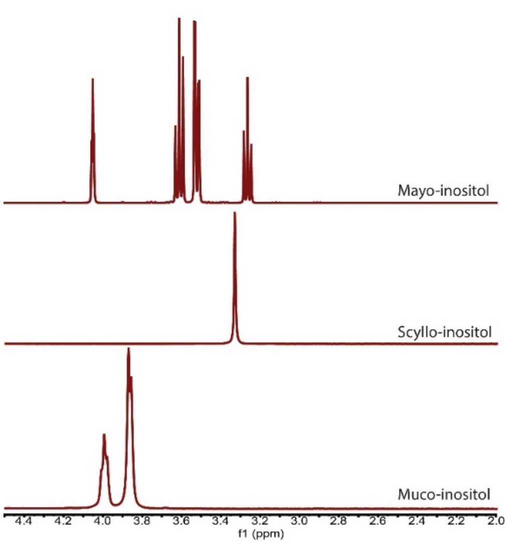

(a)

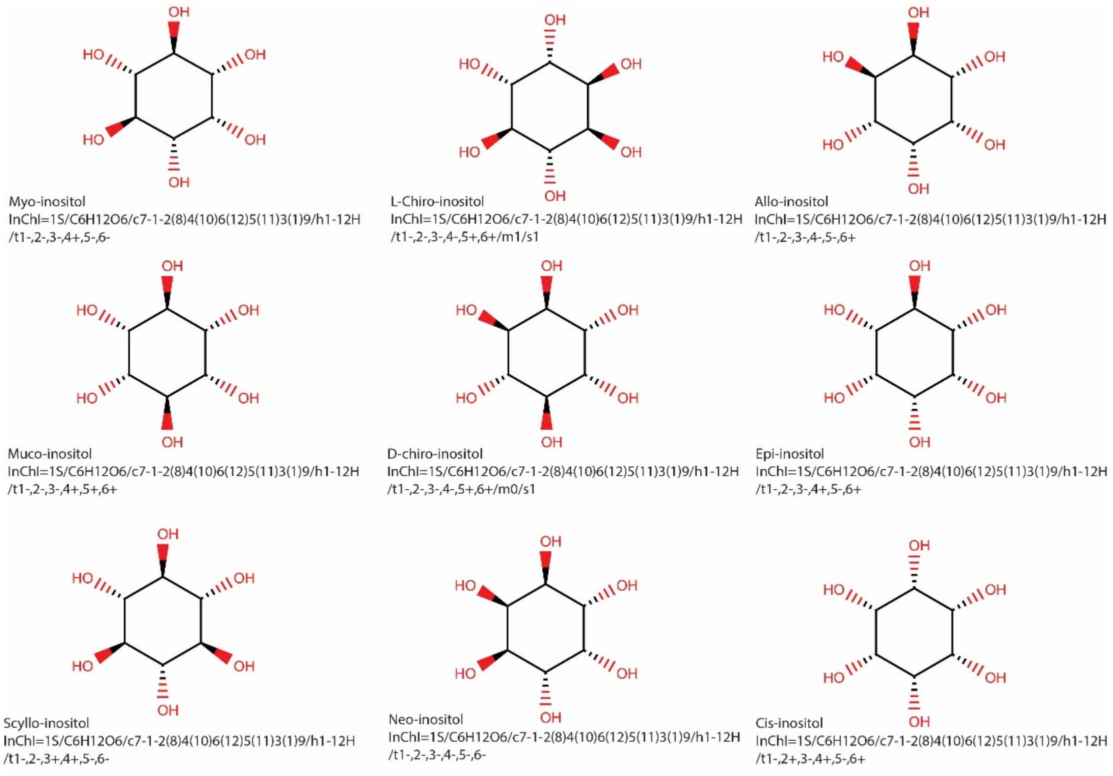

(b)

Figure 2. Importance of depositing complete standard InChI strings. (a) ^{1}H NMR spectra of the three inositol molecules as obtained from BMRB. The spectra were drawn using MestreNova v 10.0.1 (http://mestrelab.com/). (b) The nine distinct conformations of inositol family and their corresponding standard InChI strings. The differences in the stereochemistry layers of these identifiers demonstrate the importance of using complete standard InChI strings.

II. ALATIS analysis demonstrates the need for standardization of unique compound identifiers and atom labels

By comparing the standard molecule identifiers of the entries in BMRB and HMDB, we identified 701 compounds with entries in both databases. The atom labels of the paired entries matched in only 149 cases (the complete list of these molecules are provided on our website). The high percentage (78%) of incompatible atom labels highlights the need for producing unique atom identifiers. In addition to generating unique InChI strings for compounds ALATIS addresses the requirement for unique and reproducible labeling of all atoms in compounds. While the process of unique labeling of the heavy atoms of a compound is a straightforward procedure when a standard InChI string is available, the information provided in the InChI strings must be augmented in order to accommodate the reproducible labeling of the hydrogen atoms (see the Methods section for details). The main challenge in unique labeling concerns the hydrogen atoms in methylene and primary amide groups. Unique labeling is important because these hydrogen atoms can have different chemical and spectroscopic properties. In order to address this need, our approach augments standard InChI strings with consideration of the chirality of these groups to generate unique labels for all atoms of molecules.

Our software package ALATIS, takes as input the covalent structure of the compound as represented by the structure file and generates a standard InChI string and unique atom identifiers. The standard InChI identifiers for all compounds currently in BMRB and HMDB and their cross referenced compounds in PubChem are available from the ALATIS website (http://alatis.nmrfam.wisc.edu/); these identifiers enable the unique labeling of all atoms in each compound. Figure 3 shows an example of how ALATIS can be used to convert the different atom numbering schemes used by the HMDB and BMRB to a unique and reproducible atom nomenclature.

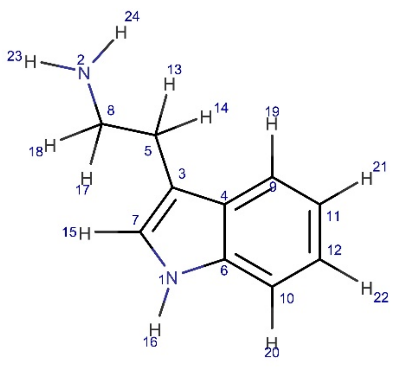

(a) Atom labeling in the HMDB entry

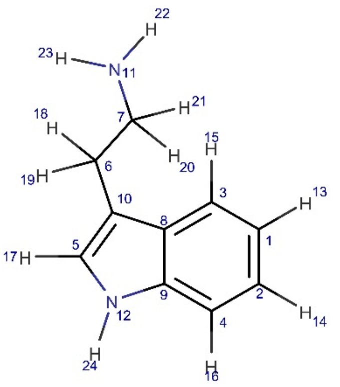

(b) Atom labeling in the BMRB entry

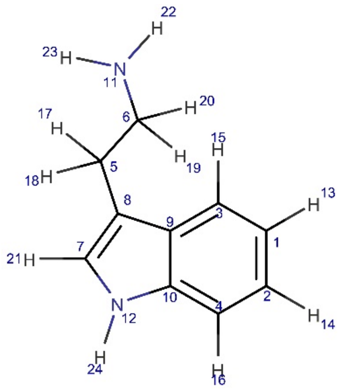

(c) Atom labeling by ALATIS for the HMDB entry

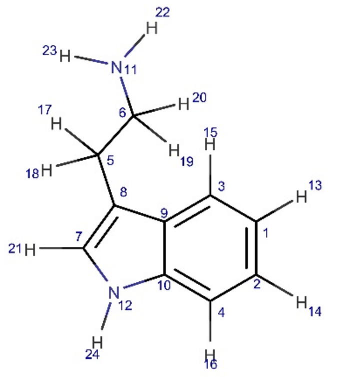

(d) Atom labeling by ALATIS for the BMRB entry

Figure 3. Unique atom label assignment using ALATIS. The 3D structure files for tryptamine were downloaded directly from the HMDB (HMDB00303) and BMRB (bmse000207) webpages. Their 2D representations are shown here. The atom labeling in HMDB and BMRB are shown, respectively, in (a) and (b). The atom labeling produced by ALATIS from the HMDB and BMRB structure files are shown, respectively in (c) and (d). ALATIS enables unique and reproducible labeling of both heavy atoms and hydrogen atoms.

III. Use of ALATIS to validate cross references

The standard InChI strings produced by ALATIS can be used to verify the correctness of existing cross references between databases. We used the output generated by ALATIS for the total of 60160 entries present in the targeted databases to identify incorrect cross references between BMRB and PubChem and between HMDB and PubChem. The standard InChI identifier of the corresponding compound was used as the frame of reference, and a cross reference was flagged as incorrect whenever there were discrepancies between the identifiers. Table 2 summarizes the statistics of these comparisons; the full list of flagged cross references is provided on our website. Analysis of the flagged cross-references identified problems in entries in all three databases. The reasons for “flagging” cross references fall into five categories: (a) incorrect mappings of entries, (b) lack of 3D structure files, (c) inconsistencies between structure files, (d) mixtures of conformers, and (e) outdated cross references. The complete list of cross references flagged under each category is provided on the ALATIS website. Specific examples are discussed below.

Table 2. Analysis of cross references between target databases. Outcomes of comparing standard InChI strings of cross references from BMRB and HMDB to PubChem entries.

	Number of cross references to PubChem	Number of incorrect cross references
BMRB	996	124
HMDB	16887	3729

(a) Incorrect mapping between data entries

The primary reason for this type of cross reference mismatch is human error. An example, is confusion between the PubChem compound and substance databases. The BMRB entry for L-arginine (bmse000711.str) mistakenly references the PubChem compound ID (CID) instead of the PubChem substance ID (SID). This error resulted in a PubChem cross reference to flopropione (PubChem CID 3362), rather than to L-arginine (PubChem SID 3362). A similar error cross referenced 4-hydroxybenzaldehyde (bmse000582) to leukotriene B3 (PubChem CID 3906), instead of citing PubChem SID 3906. Errors can occur when unique compound identifiers are not used to link two databases. For example, furan in HMDB (HMDB13785) is linked to sucrose in PubChem (PubChem CID 5988); and heparin in HMDB with the chemical formula $C_{26}H_{41}NO_{34}S_4$ (HMDB01394) is linked to the PubChem entry for STL134875 with the chemical formula $C_{18}H_{13}FN_2O_4$ (PubChem CID 46507594).

(b) Lack of 3D structure files

A major cause of flagged cross links was a missing or inaccurate 3D structure file in one of the database entries. For example, because the structure file in the HMDB entry for annosquamosin (HMDB31137) contained 2D data only, the generated InChI string lacked the stereochemistry layer present in the standard InChI generated from the PubChem entry (PubChem CID 469215).

Similarly, several flagged cross references from BMRB to PubChem were the result of absent 3D structure files in PubChem. As an example, the BMRB entry for coenzyme A (bmse000271) has a 3D structure file that produces a complete standard InChI string with a stereochemistry layer that specifies the chirality of the atoms (http://alatis.nmrfam.wisc.edu/examples/BMRB/bmse000271/). This entry is linked to a compound in PubChem (PubChem CID 6816), which reports that its 3D structure file is missing because "Conformer generation is disallowed since too flexible". The PubChem entry contains a 2D structure file, which resulted in an InChI string with an incomplete stereochemistry layer. The reason for exclusion on the basis of flexibility is unclear, because other PubChem entries contain 3D structures of flexible molecules (for example, cyclohexane (PubChem CID 8078) and arginine (PubChem CID 6322)). The PubChem entry for DL-selenomethionine (PubChem CID 15103) with the formula of $C_5H_{11}NO_2Se$ furnishes a further example. The reason for the missing 3D structure is reported to be "Conformer generation is disallowed since MMFF94s unsupported element". The corresponding 3D structure for the molecule was reported by BMRB (bmse000291), but the absence of a 3D structure in PubChem caused ALATIS to flag the cross reference. The link between arginine-glutamate complex (BMRB bmse000194) and the corresponding PubChem entry (PubChem CID 20317) was flagged because the latter lacked a 3D structure file: the 3D

structure file was reported missing because “Conformer generation is disallowed since mixture or salt”. This is an unfortunate proscription, and the rule is not always followed. One counter example with a structure file for a mixture is the PubChem entry for DL-glutamic acid (PubChem CID 611); however, the structure file, instead of specifying molecular representations for both D- and L-glutamic acid, represents the D-form alone.

(c) Inconsistency between structure files

(1) Discrepancy between number of atoms

Several cross references were flagged as the result of differences in the number of atoms in the corresponding standard InChI strings. One example of this type of problem is furnished by the cross reference from the BMRB entry for argininosuccinic acid (bmse000959) to the same compound in PubChem (CID 16950). The standard InChI generated from the BMRB structure file indicated the molecular formula (C10H19N4O6) with 19 hydrogen atoms, whereas that generated from the PubChem entry matched the molecular formula (C10H18N4O6) with 18 hydrogen atoms. The InChI-1 program reported a warning while processing the structure file of the BMRB entry that indicated a violation of a valance bond in the structure file. From the 2D representations of the structure files (shown in Figure 4), it is clear that the double bond between N10 and N12 caused violation at N12 in BMRB structure. We traced the discrepancy to a difference in the structure files in BMRB and PubChem. The structure file in the BMRB entry was a defective one downloaded from PubChem, whereas the structure file in the PubChem entry had been corrected subsequent to the download to BMRB. The InChI-1 program compensated for the violation in the BMRB structure by adding one hydrogen atom to the structure. The correct structure is that in the PubChem entry, and the BMRB entry was found to require remediation.

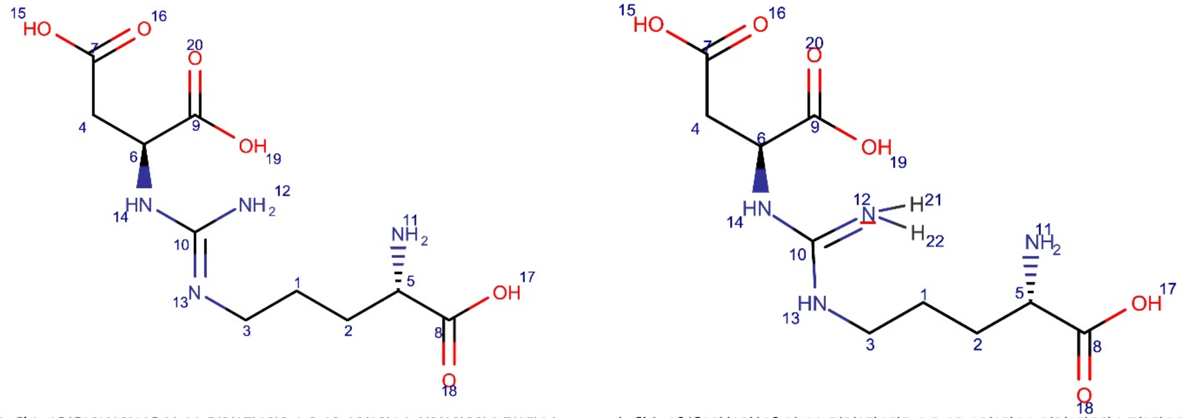

InChI=1S/C10H18N4O6/c11-5(8(17)18)2-1-3-13-10(12)14-6(9(19)20)4-7(15)16/h5-6H,1-4,11H2,(H,15,16)(H,17,18)(H,19,20)(H3,12,13,14)/t5-,6-/m0/s1

(a)

InChI=1S/C10H19N4O6/c11-5(8(17)18)2-1-3-13-10(12)14-6(9(19)20)4-7(15)16/h5-6,13-14H,1-4,11-12H2,(H,15,16)(H,17,18)(H,19,20)/t5-,6-/m0/s1

(b)

Figure 4. Identifying incorrect structure file using a unified standard identification of molecules. (a) 2D representation of the structure file from PubChem (CID 16950) and its corresponding InChI string generated using InChI-1 program. (b) 2D representation of the structure file for the same molecule from BMRB (bmse000959) and its InChI string. The error was traced to the structure file from BMRB, which had been downloaded from PubChem prior to that structure file in PubChem having been corrected. Atoms in these structures were labeled by ALATIS.

(2) Discrepancy in the InChI mirror layer ("/m")

The mirror layer in a standard InChI string can be used to process the stereochemistry layer of the string. There are cases of flagged cross references in which the only difference between the identifiers is in the mirror layer. One example of this type of flagged cross reference, is that between the BMRB entry for 2-hydroxyoctanoic acid (bmse000572) and PubChem (PubChem CID 94180). Because the structure files represent different stereochemistry (R and S isomers), the InChI-1 program generated InChI strings that differ only in their "/m" layers. The 2D representation of the 3D structure files and their corresponding standard InChI strings are shown in Figure 5.

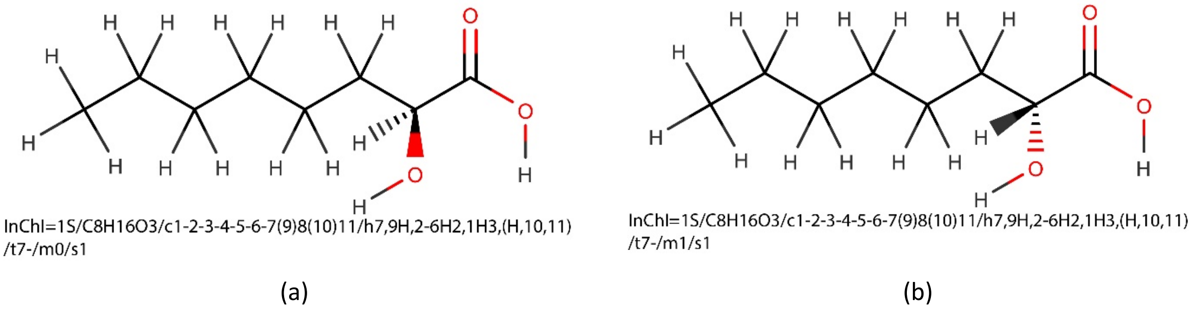

InChI=1S/C8H16O3/c1-2-3-4-5-6-7(9)8(10)11/h7,9H,2-6H2,1H3,(H,10,11)/t7-/m0/s1

(a)

InChI=1S/C8H16O3/c1-2-3-4-5-6-7(9)8(10)11/h7,9H,2-6H2,1H3,(H,10,11)/t7-/m1/s1

(b)

Figure 5. Standard InChI strings for R and S isomers. (a) 2D representation generated from the 3D structure file from PubChem (CID 94180) and its corresponding standard InChI string. (b) 2D representation generated from the 3D structure file from BMRB (bmse000572) and its generated standard InChI string. As indicated in the "/m" layer of the InChI strings, the compounds are different isomers.

(3) Discrepancy in the InChI stereochemistry layer ("/t")

The stereochemistry layer in the InChI string ("/t") represents the chirality of atoms, with +/- signs indicating the upward/downward directions. Therefore, differences in the stereochemistry layer indicate differences in chirality in the corresponding structure files. For example, the BMRB entry for N-acetyl-D-glucosamine 1-phosphate (bmse000163) cites three PubChem entries; 2-acetamido-2-deoxy-1-O-phosphonohexopyranose (PubChem CID 900), alpha-GlcNAc-1-P (PubChem CID 440364), and N-Acetyl-D-glucosamine 1-phosphate (PubChem CID 440272). Figure 6 shows 2D representations of these molecules accompanied with their corresponding standard InChI strings. As indicated in this figure, the only PubChem compound that has the same standard InChI identifier as the BMRB entry, is PubChem CID 440364 (Figure 6 b), whereas the other referenced PubChem entries have distinct stereochemistry layers and should not have been cross referenced by the BMRB entry.

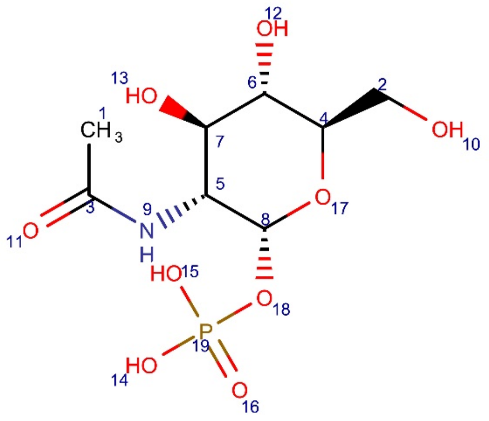

InChI=1S/C8H16NO9P/c1-3(11)9-5-7(13)6(12)4(2-10)17-8(5)18-19(14,15)16
/h4-8,10,12-13H,2H2,1H3,(H,9,11)(H2,14,15,16)/t4-,5-,6-,7-,8-/m1/s1

(a) BMRB (bmse000163) structure file

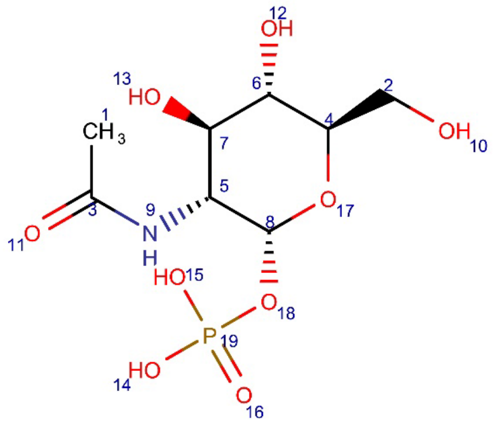

InChI=1S/C8H16NO9P/c1-3(11)9-5-7(13)6(12)4(2-10)17-8(5)18-19(14,15)16
/h4-8,10,12-13H,2H2,1H3,(H,9,11)(H2,14,15,16)/t4-,5-,6-,7-,8-/m1/s1

(b) PubChem (CID 440364) structure file

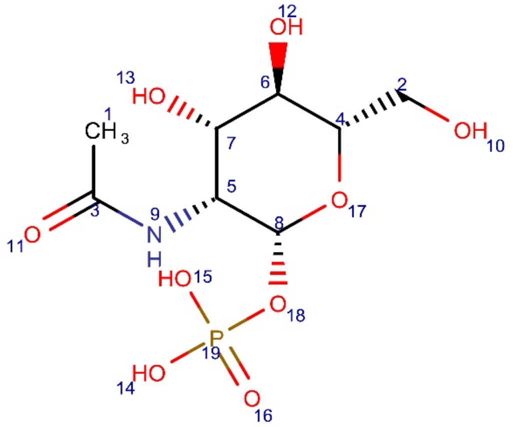

InChI=1S/C8H16NO9P/c1-3(11)9-5-7(13)6(12)4(2-10)17-8(5)18-19(14,15)16
/h4-8,10,12-13H,2H2,1H3,(H,9,11)(H2,14,15,16)/t4-,5+,6-,7-,8+/m0/s1

(c) PubChem (CID 900) structure file

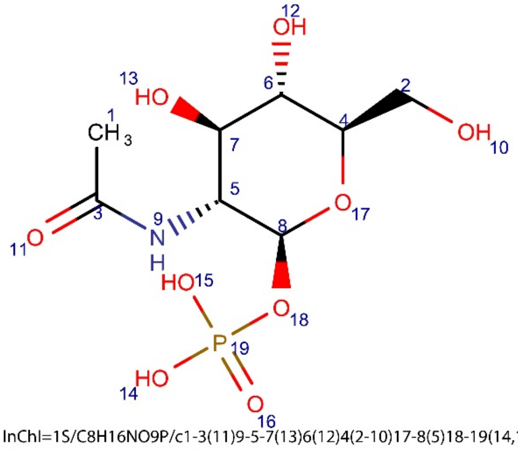

InChI=1S/C8H16NO9P/c1-3(11)9-5-7(13)6(12)4(2-10)17-8(5)18-19(14,15)16
/h4-8,10,12-13H,2H2,1H3,(H,9,11)(H2,14,15,16)/t4-,5-,6-,7-,8+/m1/s1

(d) PubChem (CID 440272) structure file

Figure 6. Differences in stereochemistry layers. The 2D representations were generated from the 3D structure files of the corresponding entries in the databases. The InChI strings for these structure files were generated by ALATIS using the InChI-1 program, and the atoms were labeled by ALATIS. The InChI string of the BMRB entry (a) matches with the InChI string of the PubChem entry (CID 440364) in (b). The structures in (c) and (d) represent different compounds in terms of their stereochemistry but were incorrectly linked to the BMRB entry.

(4) Discrepancy in the InChI isotope layer ("/i")

In the standard InChI string, the isotope layer represents atom-specific isotopic labeling of the molecules. For example, ^{12}C atoms have the highest natural abundance and are the default isotope for carbon. The occurrence of other isotopes of carbon is indicated by the isotope layer in a standard InChI string. Unexpected isotope labeling in reference compounds in databases can lead to confusion. For example, the BMRB entry for (+/-) nicotine (bmse000105) references PubChem entry AC1L41LR (CID: 175605). The corresponding structure files of these entries represent the S configuration of the molecule; however, the standard InChI string for the PubChem entry specifies ^{14}C at one of the carbon atoms leading to the mismatch flagged by ALATIS. Figure 7 shows the 2D representations and the standard InChI for these entries.

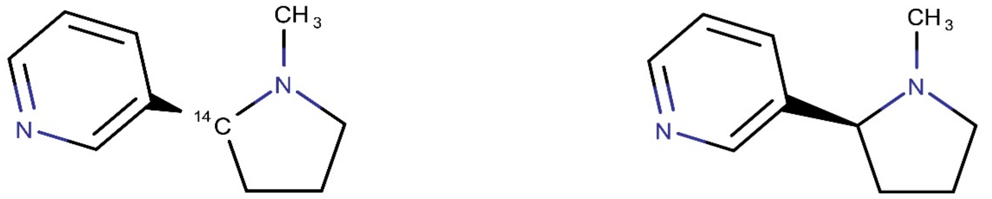

InChI=1S/C10H14N2/c1-12-7-3-5-10(12)9-4-2-6-11-8-9 /h2,4,6,8,10H,3,5,7H2,1H3/t10-/m0/s1/i3+2

(a)

InChI=1S/C10H14N2/c1-12-7-3-5-10(12)9-4-2-6-11-8-9 /h2,4,6,8,10H,3,5,7H2,1H3/t10-/m0/s1

(b)

Figure 7. Representation of isotope labeling in structure files and standard InChI strings. (a) Structure file and standard InChI string for PubChem entry CID 175605. (b) Structure file and standard InChI string for BMRB entry bmse000105. The standard InChI strings were generated by ALATIS using the InChI-1 program. The ^{14}C isotope is indicated in the "/i" layer of the InChI string in (a).

(5) Discrepancy in cis- and trans-configurations

The "/b" layer of the standard InChI string represents orientations around double bonds, namely cis and trans configurations of the molecule. The cross reference between the BMRB entry for citraconic acid (bmse000199) and PubChem (PubChem CID 874) was flagged because the molecules in the two entries differ by configuration about a double bond. As shown in Figure 8, the 3D structure files of these entries represent two different configurations, as confirmed by differences in the "/b" layer of the standard InChI identifiers.

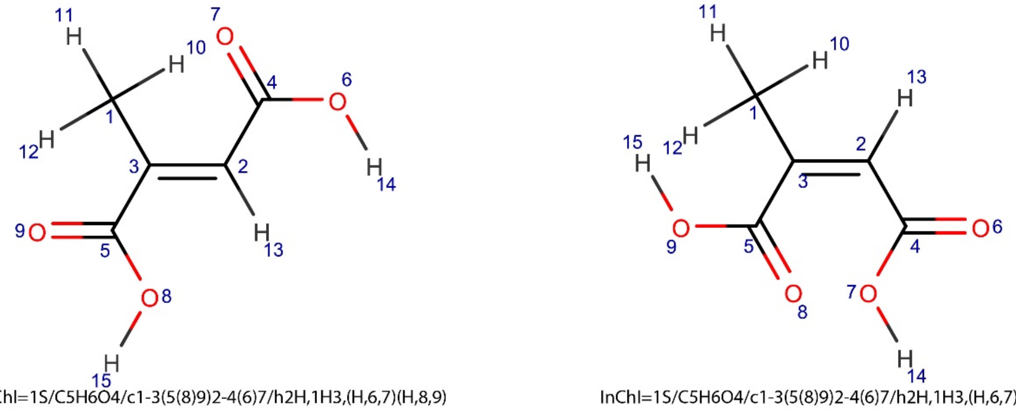

InChI=1S/C5H6O4/c1-3(5(8)9)2-4(6)7/h2H,1H3,(H,6,7)(H,8,9) /b3-2+

(a)

InChI=1S/C5H6O4/c1-3(5(8)9)2-4(6)7/h2H,1H3,(H,6,7)(H,8,9) /b3-2-

(b)

Figure 8. Representation of cis and trans-configurations in standard InChI strings. (a) Structure of the compound in PubChem (CID 874) and its standard InChI string. (b) Structure from the BMRB entry for citraconic acid (bmse000199) and its standard InChI string. The standard InChI strings were generated by ALATIS using the InChI-1 program, and the atoms were labeled by ALATIS. The stereochemistry around the the double bond C2=C3 resulted in differences in the "/b" layers of the identifiers.

(d) Mixtures of conformers

Although this category is somewhat similar to structure file inconsistency, the flagged discrepancies have significant consequences, and for this reason it is useful to treat it as a separate group. It is well-known that some molecules in solution at equilibrium can exist in more than one configuration. The 3D structure of each configuration can be accurately represented by a distinct InChI string, but if this is not done correctly, the unique identifier is compromised. This, in turn, leads to problems in the creation and validation of cross references between entries in databases. A good example of this problem is furnished by D-glucose, which is known to exist in six tautomeric states at equilibrium [3]. The BMRB entry for D-(+)-glucose (bmse000791) is linked to two PubChem entries: that for alpha-D-glucose (PubChem CID 79025), and that for (2R,3S,4R,5R)-2,3,4,5,6-pentahydroxyhexanal (PubChem CID 107526). In terms of the chemical properties of the compounds, the link from the BMRB entry to the open-chain conformation is correct. However, the two tautomers have distinct structures, and can therefore be represented by two different standard InChI identifiers. Figure 9 shows the 2D representation of the six D-glucose tautomers, their relative concentrations from [3], and their corresponding unique standard InChI identifiers. To avoid such problems, we recommend that databases incorporate all of the conformers into the database entry. This can be accomplished by incorporating the structures of the different tautomers into a single structure file, and by creating a single standard InChI string representing each tautomer. Figure 10 shows the 2D representation of the six D-glucose tautomers represented by a single structure file, its corresponding InChI string, and the unique atom labels generated by ALATIS.

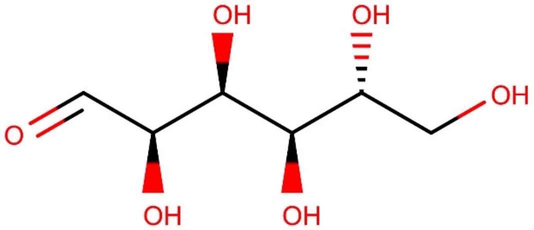

InChI=1S/C6H12O6/c7-1-3(9)5(11)6(12)4(10)2-8/h1,3-6,8-12H,2H2
/t3-,4+,5+,6+/m0/s1

(a) Glucose open chain aldehyde [0.004%]

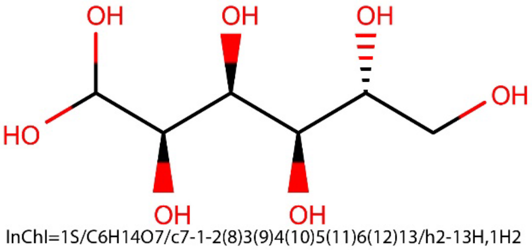

InChI=1S/C6H14O7/c7-1-2(8)3(9)4(10)5(11)6(12)13/h2-13H,1H2
/t2-,3-,4+,5-/m1/s1

(b) Glucose open chain hydrate [0.0059%]

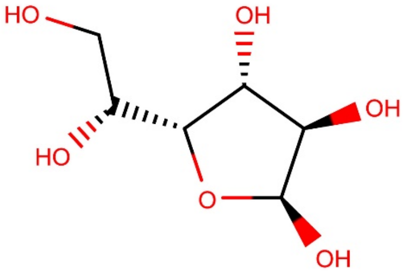

InChI=1S/C6H12O6/c7-1-2(8)5-3(9)4(10)6(11)12-5/h2-11H,1H2
/t2-,3-,4-,5-,6+/m1/s1

(c) α-furanose [0.11%]

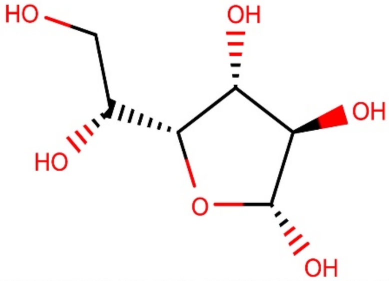

InChI=1S/C6H12O6/c7-1-2(8)5-3(9)4(10)6(11)12-5/h2-11H,1H2
/t2-,3-,4-,5-,6-/m1/s1

(d) β-furanose [0.28%]

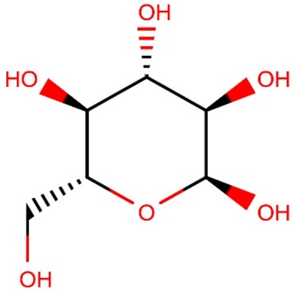

InChI=1S/C6H12O6/c7-1-2-3(8)4(9)5(10)6(11)12-2/h2-11H,1H2
/t2-,3-,4+,5-,6+/m1/s1

(e) α-glucose [37.64%]

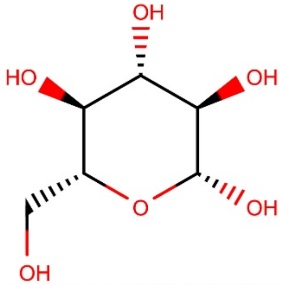

InChI=1S/C6H12O6/c7-1-2-3(8)4(9)5(10)6(11)12-2/h2-11H,1H2
/t2-,3-,4+,5-,6-/m1/s1

(f) β-glucose [61.96%]

Figure 9. 2D representations of the six D-glucose tautomers. The relative concentrations are shown in brackets; the standard InChI identifiers were generated by ATLAS using the InChI-1 program.

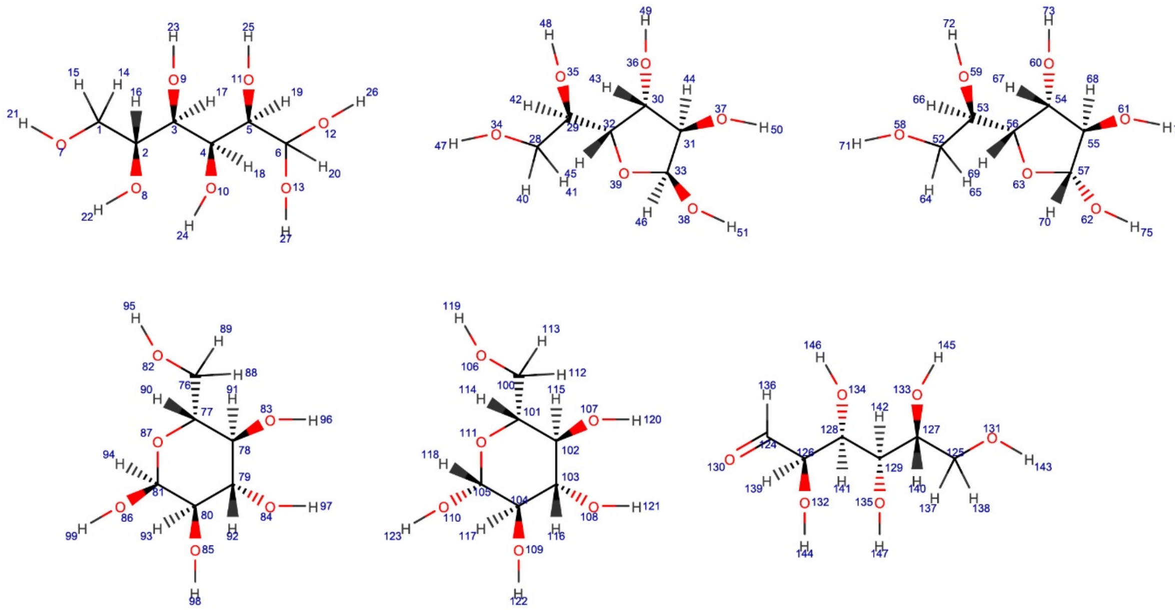

InChI=1S/C6H14O7.5C6H12O6/c7-1-2(8)3(9)4(10)5(11)6(12)13;2*7-1-2(8)5-3(9)4(10)6(11)12-5;
2*7-1-2-3(8)4(9)5(10)6(11)12-2;7-1-3(9)5(11)6(12)4(10)2-8/h2-13H,1H2;4*2-11H,1H2;1,3-6,8-12H,2H2/
t2-,3-,4+,5-;2-,3-,4-,5-,6+;2-,3-,4-,5-,6-;2-,3-,4+,5-,6+;2-,3-,4+,5-,6-;3-,4+,5+,6+/m111110/s1

Figure 10. One structure file and one standard InChI for all six D-glucose tautomers. The atoms labeled by ALATIS show the organization of the six tautomers represented in a single structure file.

(e) Obsolete data entry

This type of flagged cross reference is caused by non-systematic or incomplete database maintenance. An example of this type of error is the BMRB entry for ethanesulfonic acid (bmse000400). This entry cites two PubChem entries: the PubChem entry with CID 16211027, and the PubChem entry for ethanesulfonate (PubChem CID 3717105). The identifier for the BMRB entry matches the InChI identifier for the PubChem entry designated as ethanesulfonate. The cross reference between this entry and the PubChem entry for CID 16211027 was flagged by the ALATIS software. Manual investigation of these entries revealed that PubChem entry (CID 16211027) has an improper InChI string and is marked with “NOTE: NON-LIVE RECORD”.

IV. Use of ALATIS in creating cross references between databases

The ALATIS package produces unique and reproducible identifiers that can be used to create reliable cross references between databases. To highlight this role, we applied the procedures in ALATIS to the RCSB PDB Ligand Expo database (http://ligand-expo.rcsb.org/); RCSB PDB is a branch of the Worldwide Protein Data Bank (wwPDB)[4]. We used ALATIS to evaluate the complete set of 22758 entries (downloaded on August 18, 2016) present in this database. ALATIS was used to generate the unique compound and atoms identifiers for these compounds by deriving them automatically from the 3D structure files deposited in the Ligand Expo database. After generating the standard InChI strings and comparing them with the InChI strings deposited in the database, we detected 704 entries with non-standard InChI strings and discovered 953 entries that had InChI strings with improper stereochemistry layers. This group of compounds

was flagged by the software, and an updated database containing the unique compound and atom identifiers for these ligands was generated and made available on the ALATIS website. Next, we created the cross references by linking entries in the remediated RCSB PDB Ligand Expo database with unique standard identifiers that matched those in the ALATIS-remediated BMRB, PubChem, and HMDB databases. Reproducible uniqueness guaranteed that no compound would be missed or misidentified. As a result, we created cross references from the entries in RCSB PDB Ligand Expo to entries in the BMRB, HMDB and PubChem databases. The results are available from the ALATIS webserver.

References

1. Duchek, J., Adams, D.R. & Hudlicky, T. Chemoenzymatic synthesis of inositols, conduritols, and cyclitol analogues. *Chem Rev* **111**, 4223-4258 (2011).
2. Michell, R.H. Inositol derivatives: evolution and functions. *Nat Rev Mol Cell Biol* **9**, 151-161 (2008).
3. Bubb, W.A. NMR spectroscopy in the study of carbohydrates: Characterizing the structural complexity. *Conc Magn Reson A* **19A**, 1-19 (2003).
4. Berman, H., Henrick, K., Nakamura, H. & Markley, J.L. The worldwide Protein Data Bank (wwPDB): ensuring a single, uniform archive of PDB data. *Nucleic Acids Res* **35**, D301-303 (2007).

Supporting Information 2

Unique label assignment to all atoms

I. Unique labeling of heavy atoms

In order to assign unique labels for the heavy atoms, we first generate graph representations for the input structure file and its corresponding standard InChI string. Then, by utilizing the auxiliary outputs of the InChI-1 program, we map the elements of graphs (labeled nodes and edges), and use the mapping to assign unique labels to the heavy atoms. An illustration of these steps follows.

Constructing graph representation of heavy atoms from structure files. The structural representation of a molecule in a Molfile (or SDF file [1, 2]) consists of two main sections: atomic coordinates, and a set of connectivities (bonds) between the atoms. The atoms are labeled based on the sequence of their appearance in the lines of the first section. The second section reports bonds between atoms as referenced by the atom labels from the first section. By considering the heavy atoms as the nodes of a graph, the bonds provided in the structure file can be used to assign edges between the nodes of this graph. For example, consider the structure file of D,L-glyceraldehyde (shown in Figure 1) from BMRB (bmse000225) that represents the S configuration of the molecule. This molecule has six heavy atoms, and the process of constructing a representative graph of the heavy atoms of this molecule is shown Figure 2.

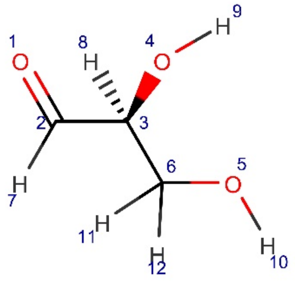

Figure 1. 2D representation of the structure file of D,L-glyceraldehyde from BMRB.

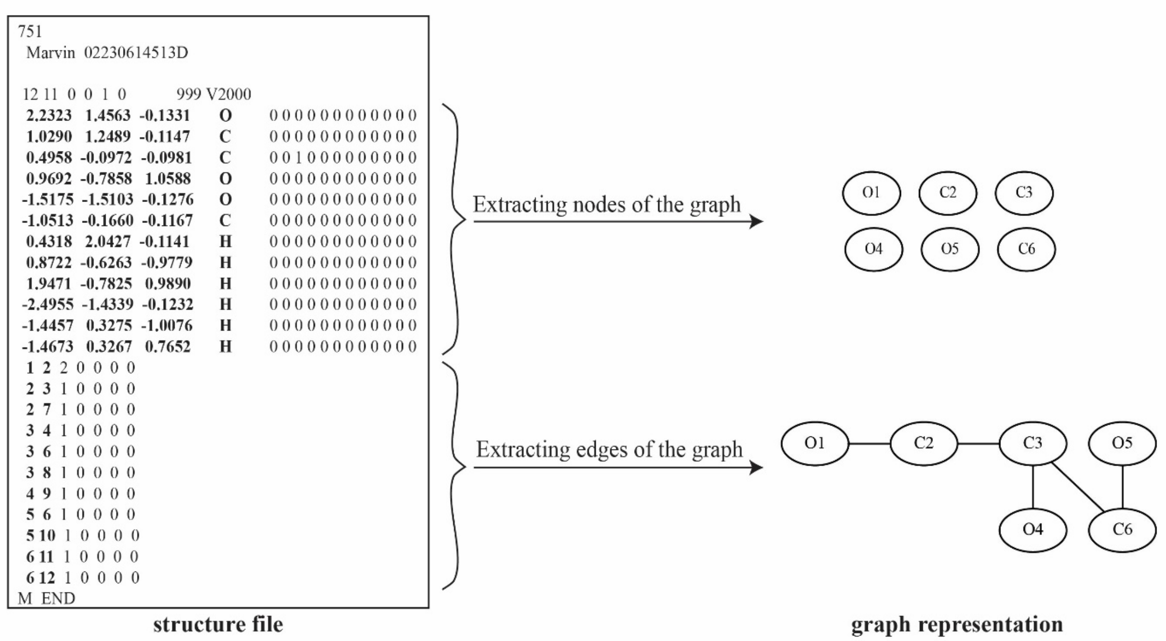

Figure 2. Constructing a graph from the heavy atoms of a structure file. The structure file, shown on left, was downloaded from BMRB entry bmse000225. The first four columns of the top section of each structure file (highlighted in bold) represents the atoms of the molecule and their corresponding coordinates. The atom labels are based on the sequence of appearance of atoms in this section. Note that the remaining columns in this section are not needed for extracting the nodes of the graph. The first two columns of the second section of the file show the bonds between the atoms. For example, the first line of this section (**1 2** 2 0 0 0 0) indicates there is a bond between the atom 1 and atom 2. These connectivies are used as the edges of the graph as shown in the graph on the right. The remaining columns of this section are not needed for extracting the edges of the graph.

Constructing graph representation of heavy atoms from standard InChI strings. By running the InChI-1 program on the input structure file, ALATIS generates a standard InChI string for the molecule. We construct a graph by parsing the connectivity information of heavy atoms contained in the InChI string. Standard InChI strings consist of different layers that are delimited by forward slashes "/". The second layer in InChI strings, which shares some similarity to the formula of the compound, is used for numbering the heavy atoms. The heavy atoms are numbered based on the sequence of their appearance in the formula (as represented by the InChI string) – the presence of hydrogens is ignored in this first stage.

Note that because standard InChI strings are unique, the sequence of atoms in the layer of the strings generated so far is unique, and therefore the numbering of the heavy atoms will remain unique. The InChI-1 program also uses the same numbering system to report the connectivity between heavy atoms in the connectivity layer ("/c")[3] of standard InChI strings. The bonds between atoms can be extracted from the "/c" layer, which results in constructing a graph for the heavy atoms. Figure 3 shows an example of this process for D,L-glyceraldehyde.

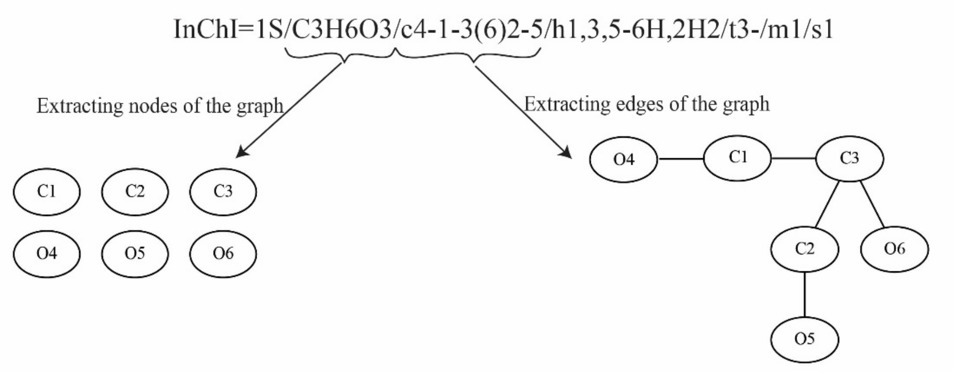

Figure 3. Constructing graph of heavy atoms from standard InChI strings. The standard InChI of D,L-glyceraldehyde was generated by the InChI-1 program. The nodes of the graph are extracted from the second layer of the InChI string, and are labeled according to their appearance in this layer. The connectivity layer of InChI strings provides the edges of the graph.

Mapping graphs generated from structure files and their corresponding InChI strings. We use the mapping information provided in the "/N" layer of the auxiliary information (AuxInfo) generated by the InChI-1 program to map the two connectivity graphs (one from the input structure file and the other from the standard InChI string of the input file). In terms of computational complexity of this process, because the mapping information is generated during the construction of the standard InChI string, the map between the graphs is a linear process. An example of the process used in mapping the graphs of heavy atoms is shown in Figure 4.

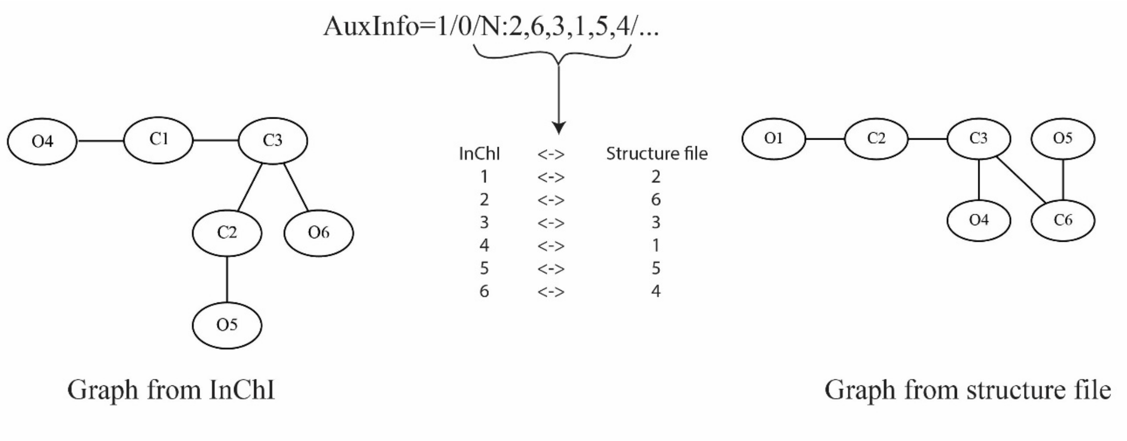

Figure 4. Mapping graph representations of a molecule. The auxiliary outputs of the InChI-1 program provide a mapping between the labels of the input structure file and the ones used in the InChI string. For this mapping we use the "/N" layer of the AuxInfo; its remaining layers are not needed for this purpose. For example, based on this map, the node labeled 1 in the InChI graph corresponds to the node labeled 2 in the graph from the structure file.

II. Unique labeling of hydrogen atoms

In order to label the hydrogen atoms, we use precedence labeling – that is, hydrogens with lower numbers are attached to a heavy atom with a lower number. Therefore, to map and label the hydrogen atoms, our first step involves the determination of the number of hydrogen atoms attached to every heavy atom. For compounds without facile exchangeable (mobile) hydrogens, the number of hydrogens attached to heavy atoms can be extracted from the "/h" layer of the InChI string [3]. However, when the compound contains mobile hydrogens, the standard InChI string representation uses possible bonds between the hydrogens and heavy atoms to delineate mobile hydrogens. In these cases, the "/h" layer of InChI is not sufficient for identifying the number of hydrogens attached to the heavy atoms. Alternatively, the mobile hydrogens are taken as fixed in the input structure file. Therefore, the number of hydrogens attached to each of the heavy atoms can be extracted from these files by means of the map generated between the graphs of the heavy atoms. This procedure enables us to label the hydrogen atoms according to the label of their corresponding heavy atoms.

After assigning unique labels to all atoms, the next issue we address is to uniquely label the hydrogen atoms in methylene and primary amide groups. These hydrogens have distinct chemical properties and require labeling in a unique and unambiguous manner. Figure 5 provides an example of a methylene group: the two hydrogen atoms directly bonded to carbon C5 of L-aspartic acid have distinct chemical shifts and can be numbered in two different ways (13 and 14 or 14 and 13).

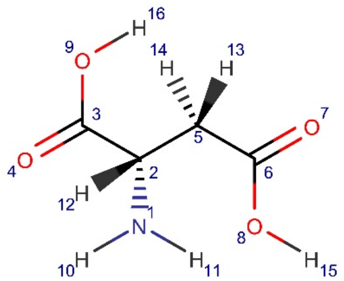

Figure 5. 2D representation of L-aspartic acid. The figure was generated from the structure file of BMRB entry bmse000031. According to this entry, hydrogen atoms 13 and 14 were assigned ambiguously to chemical shifts 2.786 and 2.703 ppm.

(a) Unique labeling of hydrogen atoms in methylene centers

To be able to match data from different databases the labels of atoms from methylene and primary amide groups must be unique and reproducible. Because the InChI-1 program considers the chirality of heavy atoms, we impose a new chirality on the heavy atoms of these groups by substituting one of the H atoms by D and then analyzing the chirality of the imposed chiral center. This substitution is performed in the background by modifying the input structure file of the compound such that after labeling the hydrogen atoms the modified structure file will be restored to its original form. After running the InChI-1 program on the modified structure file, the substituted atom may or may not force a new chirality layer ("/t") in the InChI string. If

substitutions of none of the hydrogen atoms imposes any new chirality, it means the hydrogen atoms are indistinguishable, and therefore there is no priority on numbering the atoms. However, when the substitution imposes a new chirality, the corresponding InChI string provides an accurate representation of it. Figure 6 illustrates an example of this process.

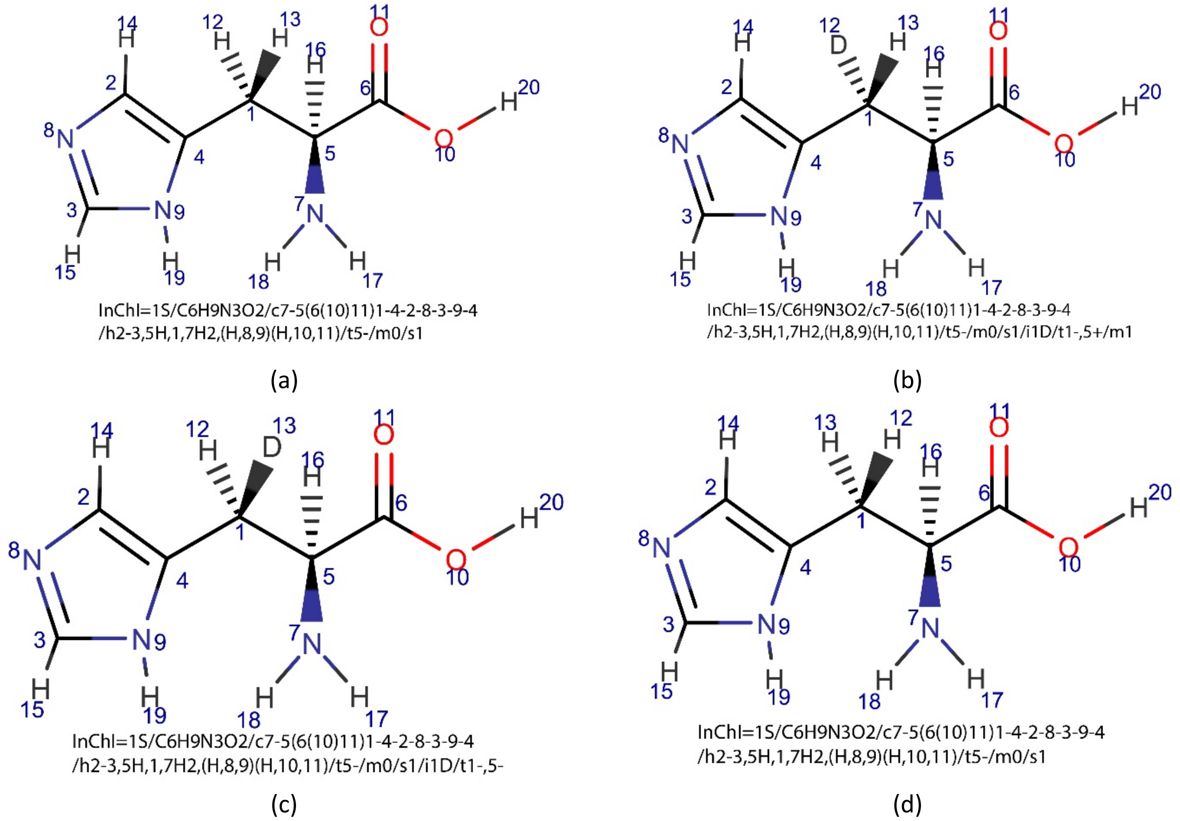

Figure 6. Procedure used in labeling the prochiral methylene hydrogen atoms connected to C1 of L-histidine. (a) The output of ALATIS with provisional labeling of L-histidine. The stereochemistry layer of the InChI string "/t5-" indicates a chiral center at C5 with a "-" sign. We note that the mirror flag is 0 ("/m0") for this InChI string. (b) By substituting the H12 to D12, the standard InChI string indicates an additional stereochemistry layer "/t1-,5+" with "/m1". The mirror flag means the "/t" layer should be read as "/t1+,5-". The stereo sign of C5 is the same as its original sign in (a), and, therefore, the substitution imposed a new chiral center at C1 with "+" sign. (c) Substitution of H13 to D13 imposed a new chiral center at C1 with a "-" sign. According to convention, the H whose substitution to D imposed a "+" stereo sign should be assigned a higher number than the H whose substitution to D imposed a "-" sign. Therefore, the provisional labels H12 and H13 should be swapped. (d) The final atom labels following swapping of the hydrogens atoms. ALATIS carries out this type of analysis automatically to generate these final atom designators.

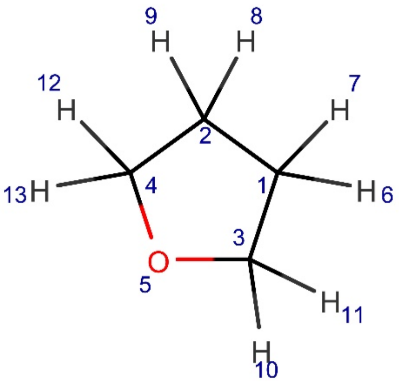

(a) InChI=1S/C4H8O/c1-2-4-5-3-1/h1-4H2

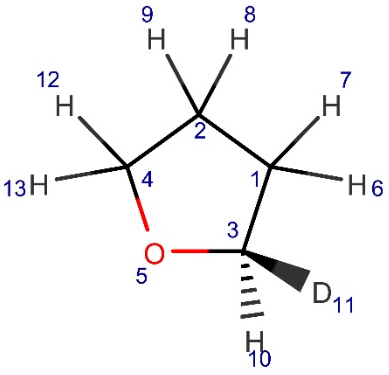

(b)

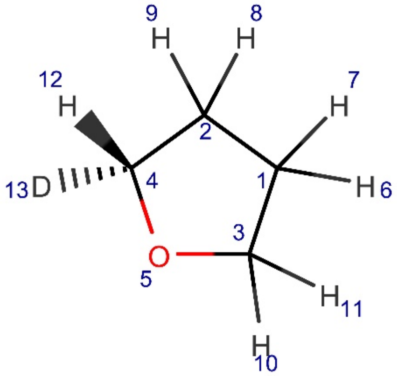

(c)

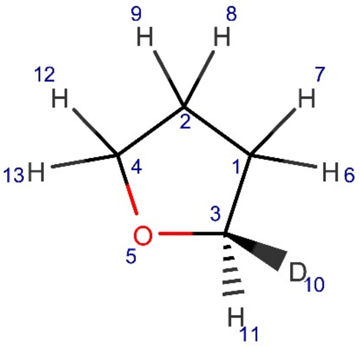

(d) InChI=1S/C4H8O/c1-2-4-5-3-1/h1-4H2/i3D/t3-/m1/s1

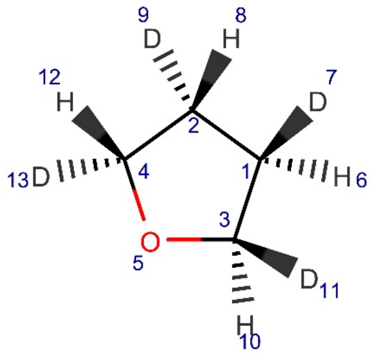

(e) InChI=1S/C4H8O/c1-2-4-5-3-1/h1-4H2/i1D,2D,3D,4D/t1-,2-,3-,4-/m1/s1

Figure 7. Effects of substituting H to D in the methylene groups of a symmetric achiral molecule. During the normalization process of the InChI-1 program, the heavy atoms are relabeled such that the generated standard InChI strings for these molecules are the same. (a) 2D representation of furan and its corresponding standard InChI string; (b) Substitution of H11 to deuterium; (c) substitution of H13 to deuterium; (d) 2D representation of the unique InChI strings of the molecules from (b) and (c); (e) 2D representation of the result of simultaneous substitution of one H to D from each of the methylene groups. The corresponding standard InChI string shows chiral centers at each carbon. These are then used in deriving unique labels for all methylene hydrogens of the molecule.

The final challenge involves methylene groups in symmetric achiral molecules. Whereas the above labeling strategy for the hydrogen atoms is practical for chiral compounds, symmetric achiral compounds require additional attention. Because of the way InChI strings are constructed, the InChI-1 program generates identical InChI strings when the H on either methylene is substituted for D. Because the molecule is achiral and symmetric, the generated InChI strings are not incorrect. For example, for the furan ring shown in Figure 7, when an H on C3 or C4 is substituted to D, the InChI-1 program generates the same InChI string "InChI=1S/C4H8O/c1-2-4-5-3-1/h1-4H2/i3D/t3-/m1/s1" for the molecule. In each case, the InChI-1 program numbers the heavy atoms such that the isotope (deuterium) is attached to the heavy atom that is labeled 3 ("/i3D"). To overcome this problem, we substitute one H of every methylene group in the compound to D simultaneously. Because of this substitution, the recently imposed chiral centers of the compound do not lead to permutation of the labels of the heavy atoms. Therefore, a unique InChI string is generated, and by considering the stereochemistry layer of this string, ALATIS assigns unique labels to the hydrogen atoms by following the process described above. Figure 7.e shows the effect of this substitution on the generated standard InChI string.

(b) Unique labeling of hydrogen atoms in primary amide centers

To assign unique labels to the hydrogen atoms of an amide center, we follow the same strategy as with methylene centers: atom substitutions that impose new chiral centers. In order to distinguish between the two hydrogen atoms of a primary amide center (R-CO-NH2), we substitute the amide group to (R-CO-CH2-SH) and then follow the process of deuterium substitution to impose new chirality on the methylene center. The NH2 to CH2-SH substitution was decided upon because the procedure leads to an effective way of uniquely labeling the hydrogen atoms of the amide group. The coordinates of the heavy atom (the N substituted C) and its hydrogen atoms remain fixed. Because this substitution changes the number of atoms in the structure file (number of nitrogen atoms reduced by 1, number carbon atoms increased by 1, and added S-H), the unique labels of the atoms need to be updated. ALATIS considers these changes in the atom labels and keeps track of the atoms (NH2), such that after assigning unique labels to the amide hydrogen atoms, the output structure file contains the unique labels for all atoms. Figure 8 shows an example of this process for L-asparagine.

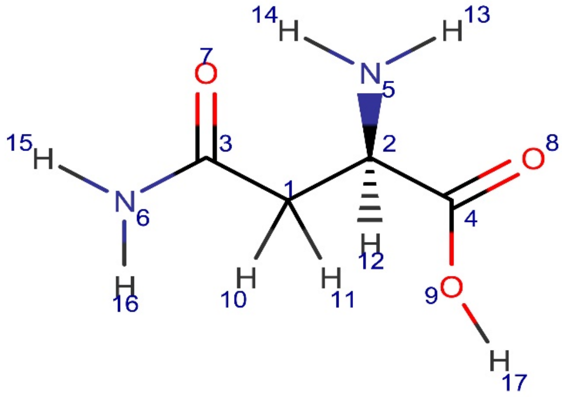

InChI=1S/C4H8N2O3/c5-2(4(8)9)1-3(6)7
/h2H,1,5H2,(H2,6,7)(H,8,9)/t2-/m0/s1

(a)

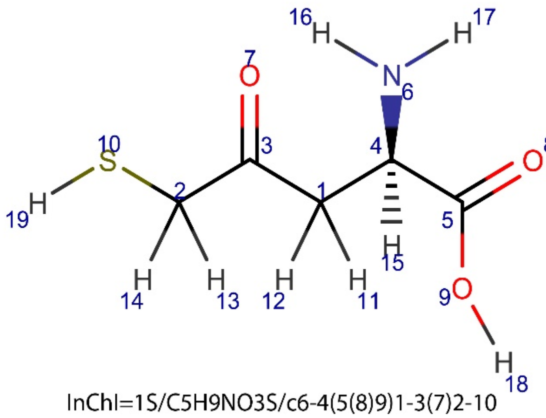

InChI=1S/C5H9NO3S/c6-4(5(8)9)1-3(7)2-10
/h4,10H,1-2,6H2,(H,8,9)/t4-/m0/s1

(b)

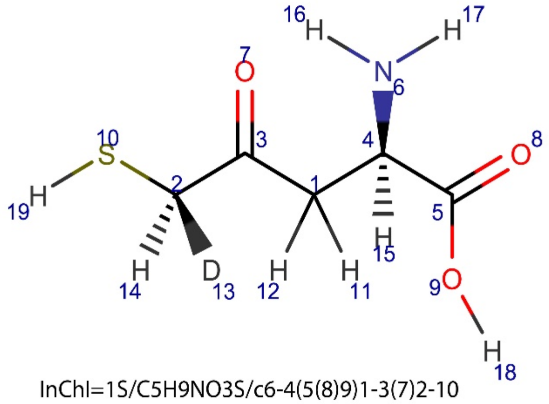

InChI=1S/C5H9NO3S/c6-4(5(8)9)1-3(7)2-10
/h4,10H,1-2,6H2,(H,8,9)/t4-/m0/s1/i2D/t2-,4+/m1

(c)

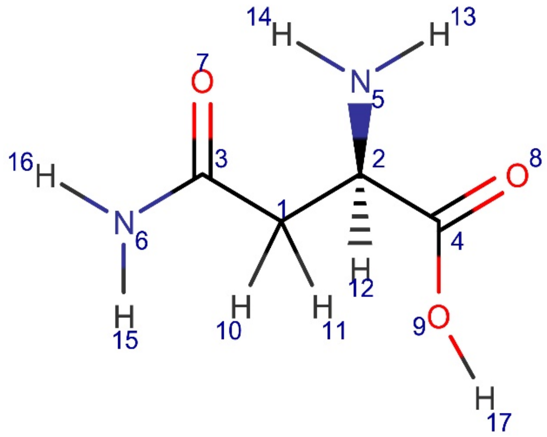

(d)

Figure 8. Unique labeling of the primary amide group of L-asparagine. (a) Preliminary output of ALATIS with atom numbering prior to processing the primary amide group. (b) 2D representation of the structure file after substituting the primary amide group to CH2-SH, and its corresponding standard InChI string. Because of the changes in the atoms (N6 to C2, H15 to H13, H16 to H14, and the additional S-H) in the molecule, ALATIS changed the unique atom labels according to the new standard InChI string. (c) Imposing a new chirality at C2 by substituting H13 to D13. The standard InChI after the deuterium substitution contains a new chirality layer "t2-,4+" with a mirror layer "/m1", which indicates the modification imposed a (+) chirality at C2. As mentioned above, the "+" chirality indicates the corresponding hydrogen atom should have a higher label than the other hydrogen atom. (d) 2D representation of the output of ALATIS after analyzing the amide group. According to the analysis in (c), the labels of the hydrogen atoms (H15 and H16) in (a) should be swapped, as indicated in this figure. Note that the primary amide analysis only affects the labels of the amide hydrogens: labels of the other atoms in (a) and (d) remain unchanged and unique.

III. Unique labeling of all atoms in a mixture of compounds

In order to assign unique atom labels to a structure file containing a mixture of molecules, ALATIS uses the aforementioned atom labeling strategies to assign unique labels to all atoms of the mixture. Then ALATIS uses the information layers in standard InChI strings [3] to delaminate and identify the individual molecules in the mixture and organizes the atom labels such that every molecule in the mixture will be associated to a block of indices. Figure 9 shows an example of this process for the BMRB entry (bmse000194), which deals with the mixture of L-arginine and L-glutamate. In this process, the organization of the molecules is based on the sequence of their appearances in the corresponding standard InChI string for the mixture. We note that since standard InChI strings are unique, the sequence of appearance of molecules in the InChI string of a mixture is unique, and therefore the arrangement of these molecules in the output of ALATIS is unique.

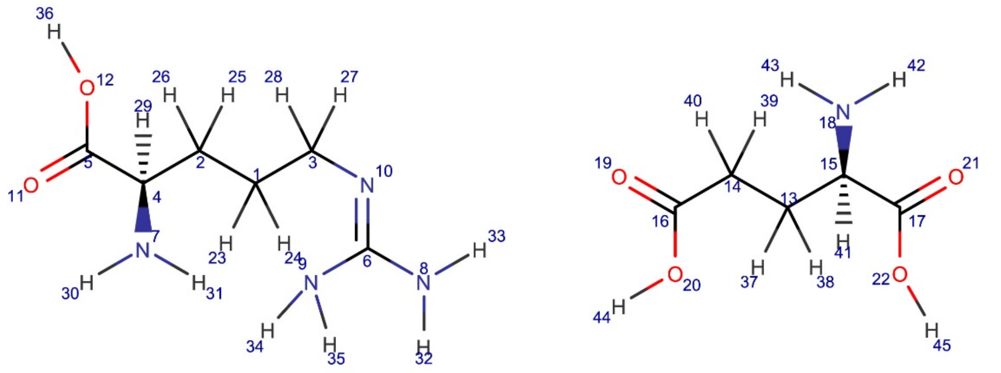

(a)

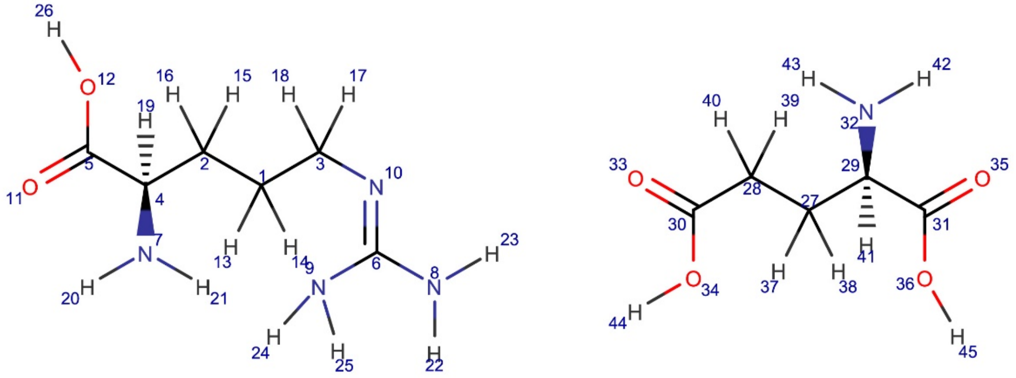

InChI=1S/C6H14N4O2.C5H9NO4/c7-4(5(11)12)2-1-3-10-6(8)9;6-3(5(9)10)1-2-4(7)8
/h4H,1-3,7H2,(H,11,12)(H4,8,9,10);3H,1-2,6H2,(H,7,8)(H,9,10)/t4-;3-/m01/s1

(b)

Figure 9. Illustration of atom label assignment in mixtures. (a) Initial labeling by ALATIS before organizing the labels in the mixture. As shown, the heavy atoms in the mixture are labeled consecutively (labels 1 to 22), and then the hydrogen atoms (23:45) in the mixture are labeled accordingly. (b) Organization of atom labels based on the appearance of the molecules in the standard InChI string. The formula layer of InChI (C6H14N4O2.C5H9NO4) organizes the molecules as L-arginine (C6H14N4O2) then L-glutamate (C5H9NO4). In the output of ALATIS, the heavy atoms and hydrogens corresponding to L-arginine are indexed first (1:26) followed by the heavy atoms and hydrogens of L-glutamate (27-45).

One example of this capability of ALATIS is the unique labeling of all atoms of the six tautomers of D-(+)-glucose that are captured in one structure file (see Results Section). Another example that illustrates the importance of this capability is the atom labeling of methylamine with two different counter ions (Figure 10).

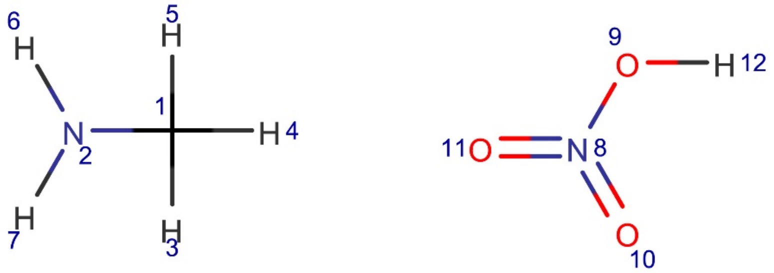

InChI=1S/CH5N.HNO3/c1-2;2-1(3)4/h2H2,1H3;(H,2,3,4)

(a)

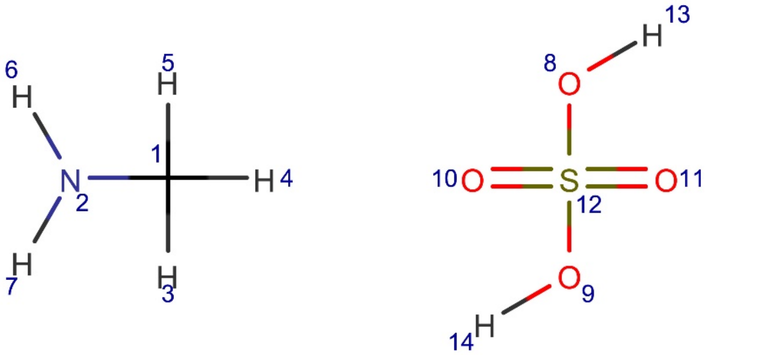

InChI=1S/CH5N.H2O4S/c1-2;1-5(2,3)4/h2H2,1H3;(H2,1,2,3,4)

(b)

Figure 10. Representation of mixtures based on standard InChI strings. (a) methylamine nitrate, (b) methylamine sulfate. Organizing the atom labels by ALATIS based on appearance of molecules in the standard InChI strings resulted in consistent atom labeling of methylamine (labels 1:7) regardless of differences in the counter ion in the structure files.

References

1. Hall, B. & Leahy, M. G. *Open Source Approaches in Spatial Data Handling*. (Springer-Verlag Berlin Heidelberg, 2008).
2 Dalby, A. *et al.* Description of several chemical structure file formats used by computer programs developed at Molecular Design Limited. *J Chem Inf Comput Sci* **32**, 244-255 (1992).
3. Downloads of InChI Software—InChI Trust. http://www.inchi-trust.org/downloads/ (Aug 2016).
